# Cell type-specific contextualisation of the human phenome: towards the systematic treatment of all rare diseases

**DOI:** 10.1186/s13073-026-01692-0

**Published:** 2026-06-26

**Authors:** Brian M. Schilder, Kitty B. Murphy, Hiranyamaya Dash, Yichun Zhang, Robert Gordon-Smith, Jai Chapman, Momoko Otani, Nathan G. Skene

**Affiliations:** 1 Department of Brain Sciences, Imperial College London, Burlington Danes Building, Hammersmith Hospital Campus, Du Cane Road, W12 0NN London, UK; 2 UK Dementia Research Institute, Imperial College London, Burlington Danes Building, Hammersmith Hospital Campus, Du Cane Road, W12 0NN London, UK; 3 National Heart and Lung Institute, Imperial College London, London, UK

**Keywords:** Rare disease, Human phenotype ontology, Single-cell transcriptomics, Cell type specificity, Phenotype-cell type associations, Gene therapy, Therapeutic target identification

## Abstract

**Background:**

Rare diseases (RDs) are a highly heterogeneous and underserved group of conditions. Most RDs have a strong genetic basis but their causal pathophysiological mechanisms remain poorly understood, limiting the development of targeted therapies.

**Methods:**

We systematically characterised the cell type-specific mechanisms underlying all genetically defined RD phenotypes by integrating the Human Phenotype Ontology (HPO) with whole-body single-cell transcriptomic atlases from embryonic, foetal, and adult samples. Associations were validated against orthogonal biomedical knowledge graphs and then prioritised by strength of supporting evidence, clinical severity, and gene-therapy compatibility.

**Results:**

We identified significant associations between 201 cell types and 9,575/11,028 (86.7%) phenotypes across 8,628 RDs, substantially expanding knowledge of phenotype-cell type links. Prioritisation by severity (e.g. lethality, motor or mental impairment) and gene-therapy compatibility (e.g. cell type specificity, postnatal treatability) identified candidate phenotypes and cell types for therapeutic targeting.

**Conclusions:**

We present a scalable, reproducible framework for phenome-wide, cell type-specific mechanism prediction in rare diseases, providing a major step toward systematic therapeutic development for patients across a broad spectrum of serious RDs.

**Software and data availability:**

Interactive web portal: https://neurogenomics-ukdri.dsi.ic.ac.uk/. R packages introduced in this study: KGExplorer (https://github.com/neurogenomics/KGExplorer), HPOExplorer (https://github.com/neurogenomics/HPOExplorer), and MSTExplorer (https://github.com/neurogenomics/MSTExplorer). Manuscript analyses and reproducibility code: https://github.com/neurogenomics/rare_disease_celltyping.

**Supplementary Information:**

The online version contains supplementary material available at 10.1186/s13073-026-01692-0.

## Background

Rare diseases (RDs) are individually uncommon but collectively this class of over 10,000 conditions affects 300–400 million people worldwide (1 in 10–20 individuals) [1, 2]. Approximately 75% of RD patients are children, with a 30% rate of mortality by age five [3]. Diagnosis is challenging due to highly variable presentations and scarcity of specialists; diagnostic odysseys last five years on average [4], with $\sim$46% of patients misdiagnosed and >75% never diagnosed [5]. Prognosis is similarly difficult. Treatments exist for <5% of RDs [6] and high development costs for small patient populations deter investment [7, 8], making these therapies among the world’s most expensive [9, 10]. High-throughput therapeutic discovery could lower costs and accelerate delivery.

A major barrier in research and clinical care of diseases is inconsistent medical terminology. The Human Phenotype Ontology (HPO) provides a unified, hierarchical framework of 18,082 phenotypes spanning 10,300 RDs [11–13], integrated into diagnostics and linked to other ontologies (e.g. SNOMED CT, UMLS, ICD). Over 80% of RDs have known genetic causes [14], with HPO gene annotations curated from OMIM, Orphanet, DECIPHER, and case reports. Yet gene lists alone lack the tissue and cell type context essential for understanding pathogenesis and improving diagnosis, prognosis, and treatment.

Single-cell RNA-seq (scRNA-seq) now enables transcriptome-wide profiling at cellular resolution [15–17]. Comprehensive atlases such as Descartes Human [18] and Human Cell Landscape [19] together span embryonic, foetal, and adult stages across tissues, providing gene signatures for hundreds of cell subtypes. Integrating RD gene annotations with these profiles reveals the specific cell types through which genes act, including understudied cell types.

Cell type-specific mechanisms are critical for guiding the development of effective therapeutics, especially virally-mediated gene therapies [20, 21]. Knowledge of the specific causal cell types can enhance efficacy and improve safety by avoiding off-target effects. To facilitate these key insights, we developed a high-throughput pipeline to nominate cell type-resolved gene therapy targets across thousands of RD phenotypes, ranked by composite phenotype severity scores [22]. This work expands knowledge of the cell types, organ systems, and life stages underlying RDs, with direct applications to precision therapeutic development.

**Fig. 1:**
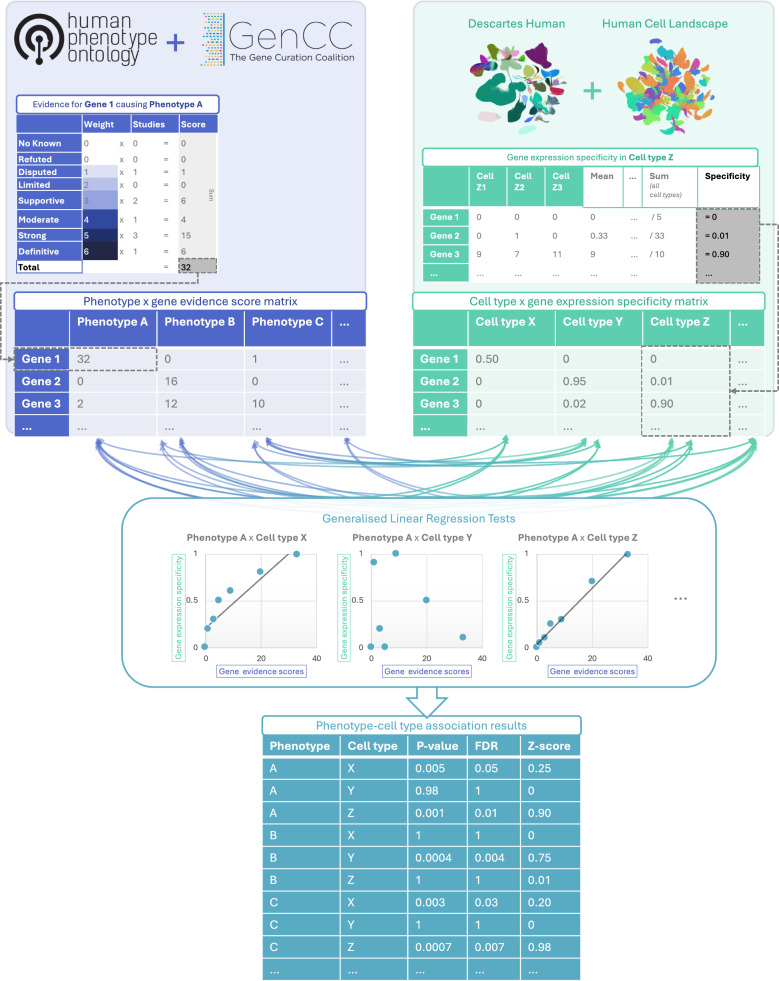
Multi-modal data fusion reveals the cell types underlying thousands of human phenotypes. Schematic overview of study design in which we numerically encoded the strength of evidence linking each gene and each phenotype (using the Human Phenotype Ontology and GenCC databases). We then created gene signature profiles for all cell types in the Descartes Human and Human Cell Landscape scRNA-seq atlases. Finally, we iteratively ran generalised linear regression tests between all pairwise combinations of phenotype gene signatures and cell type gene signatures. The resulting associations were then used to nominate cell type-resolved gene therapy targets for thousands of rare diseases

**Fig. 2:**
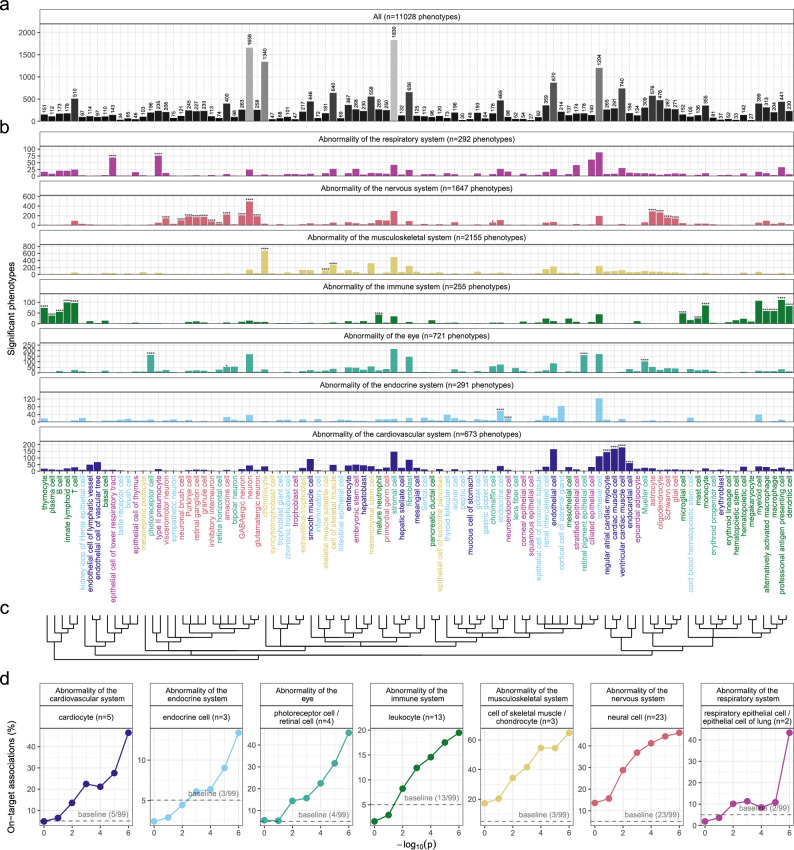
High-throughput analysis reveals cell types underlying thousands of rare disease phenotypes. **a**, Some cell types are much more commonly associated with phenotypes than others. Bar height indicates the total number of significant phenotype associations per cell type (FDR<0.05) across all branches of the HPO. **b**, Analyses reveal expected and novel cell type associations within high-level HPO branches. Asterisks above each bar indicate whether that cell type was significantly more often enriched in that branch relative to all other HPO branches, including those not shown here, as a proxy for how specifically that cell type is associated with that branch; FDR<0.0001 (****), FDR<0.001 (***), FDR<0.01 (**), FDR<0.05 (*). **c**, Ontological relatedness of cell types in the Cell Ontology (CL) [31]. **d**, The proportion of on-target associations (*y-axis*) increases with greater test significance (*x-axis*). Percentage of significant phenotype associations with on-target cell types (second row of facet labels), respective to the HPO branch

**Fig. 3:**
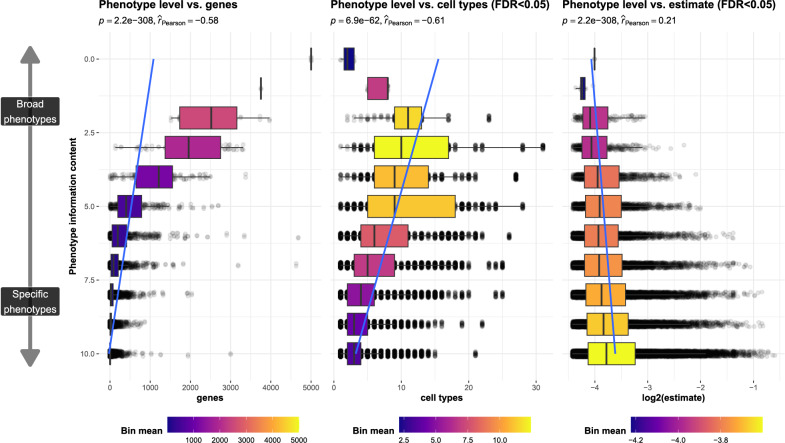
More specific phenotypes are associated with fewer, more specific genes and cell types. Information content (IC), is a normalised measure of ontology term specificity. Terms with lower IC represent the broadest HPO terms (e.g. ‘All’), while terms with higher IC indicate progressively more specific HPO terms (e.g. ‘Contracture of proximal interphalangeal joints of 2nd-5th fingers’). Box plots show the relationship between HPO phenotype IC and **a**, the number of genes annotated to each phenotype, **b**, the number of significantly enriched cell types, **c**, the effect sizes (absolute model $R^2$ estimates after log-transformation) of significant phenotype-cell type association tests. IC was binned only for the purposes of visualisation; all statistics were performed on the unbinned data. Boxes are coloured by the mean *x-axis* value within each IC bin

**Fig. 4:**
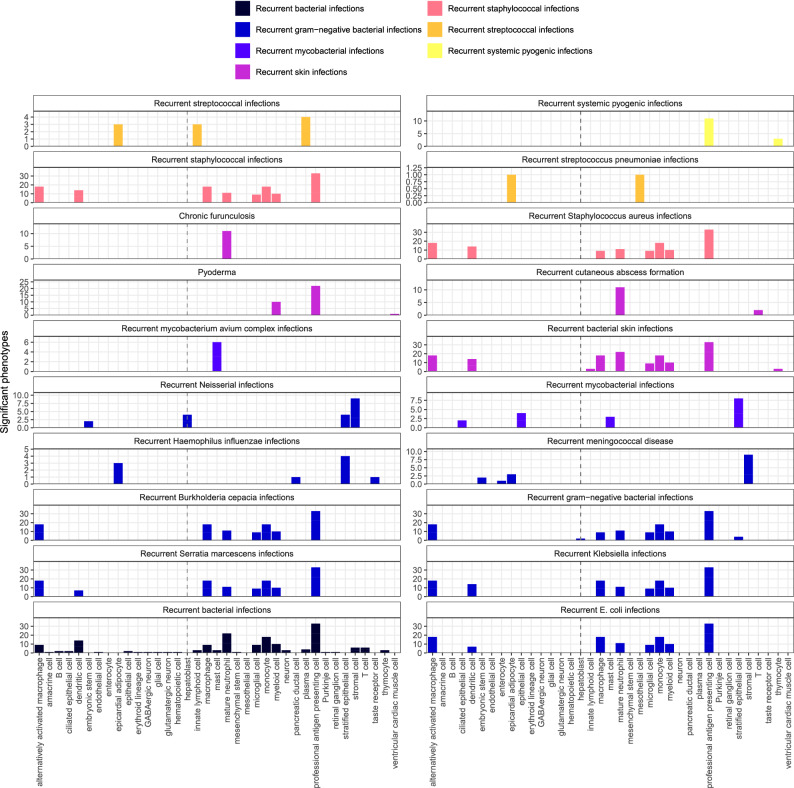
Recurrent bacterial infection subtypes are associated with different sets of immune and non-immune cell types. Significant phenotype-cell type tests for phenotypes within the branch ‘Recurrent bacterial infections’. Amongst all different kinds of recurrent bacterial infections, hepatoblasts (*highlighted by vertical dotted lines*) are exclusively associated with ‘Recurrent Neisserial infections’. Note that terms from multiple levels of the same ontology branch are shown as separate facets (e.g. ‘Recurrent bacterial infections’ and ‘Recurrent gram-negative bacterial infections’)

**Fig. 5:**
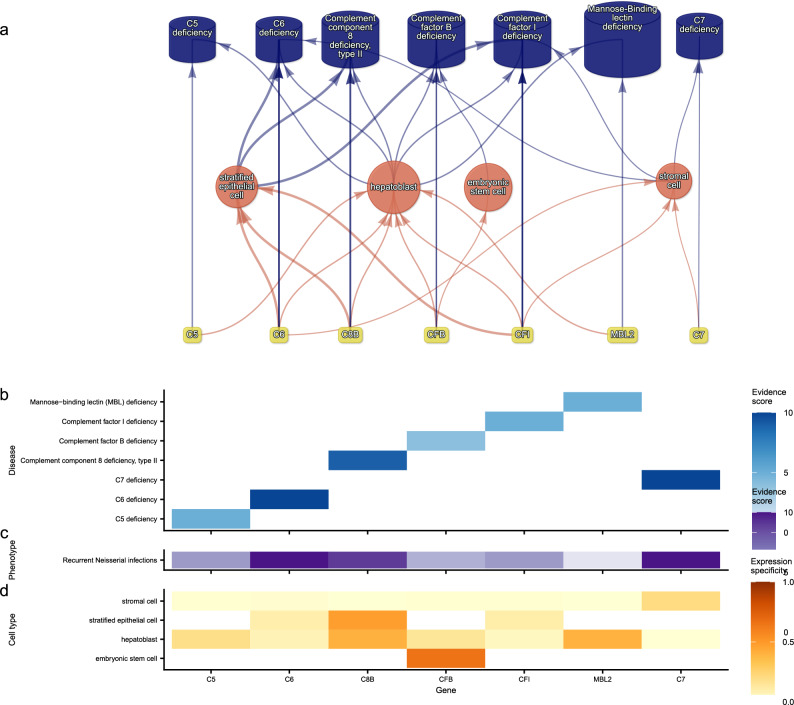
Causal network of recurrent Neisserial infections (RNI) reveals multi-scale disease etiology. RNI is a phenotype in seven different monogenic diseases caused by disruptions to specific complement system genes. Four cell types were significantly associated with RNI. **a**, One can trace how genes causal for RNI (*yellow boxes, bottom*) mediate their effects through cell types (*orange circles, middle*) and diseases (*blue cylinders, top*). Cell types are connected to RNI via association testing (FDR<0.05). Genes shown here have both strong evidence for a causal role in RNI and high expression specificity in the associated cell type. Cell types can be linked to monogenic diseases via the genes specifically expressed in those cell types (i.e. are in the top 25% of cell type specificity expression quantiles). Nodes are arranged using the Sugiyama algorithm [44]. **b** Expression specificity quantiles (1–40 scale) of each driver gene in each cell type (darker = greater specificity). **c** GenCC-derived evidence scores between the RNI phenotype and each gene. **d** Expression specificity (0 = least specific, 1 = most specific) of each gene in each cell type

**Fig. 6:**
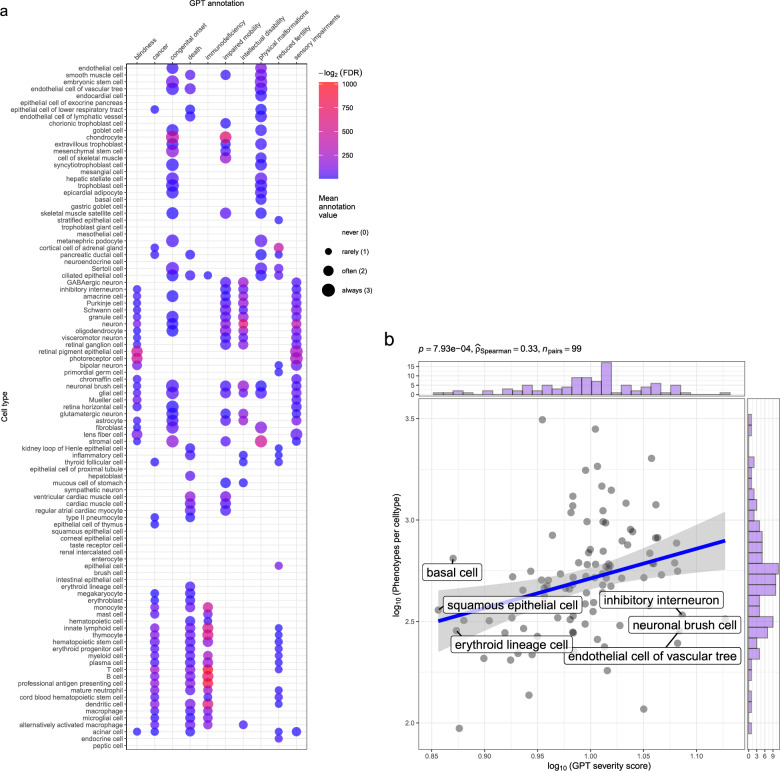
Genetic disruptions to some cell types cause more clinically severe phenotypes than others. **a**, Different cell types are associated with different aspects of phenotypic severity. The dot plot shows the mean encoded frequency value for a given severity annotation (0=“never”, 1=“rarely”, 2=“often”, 3=“always”; shown as dot size), aggregated by the associated cell type. One-sided Wilcoxon rank-sum tests were performed for each cell type (within each GPT annotation) to determine which cell types more frequently caused severe phenotypes than all other cell types. Dots are colored by $-log_2(FDR)$ when Wilcoxon test FDR values were less than 0.05. All dots with non-significant Wilcoxon tests are not shown. Cell types (rows) are clustered according to the *p*-values of the Wilcoxon tests. **b**, Cell types that affect more phenotypes tend to have more clinically severe consequences. Specifically, the number of phenotypes each cell type is significantly associated with, and the mean composite severity score of each cell type. The cell types with the top/bottom three x/y axis values are labeled to illustrate the cell types that cause the most/least phenotypic disruption when dysfunctional. Side histograms show the density of data points along each axis. Summary statistics for the linear regression are shown in the title ($p$ = *p*-value, $\hat{\rho }_{Spearman}$ = Spearman rank correlation coefficient, $n_{pairs}$ = number of observed data pairs)

**Fig. 7:**
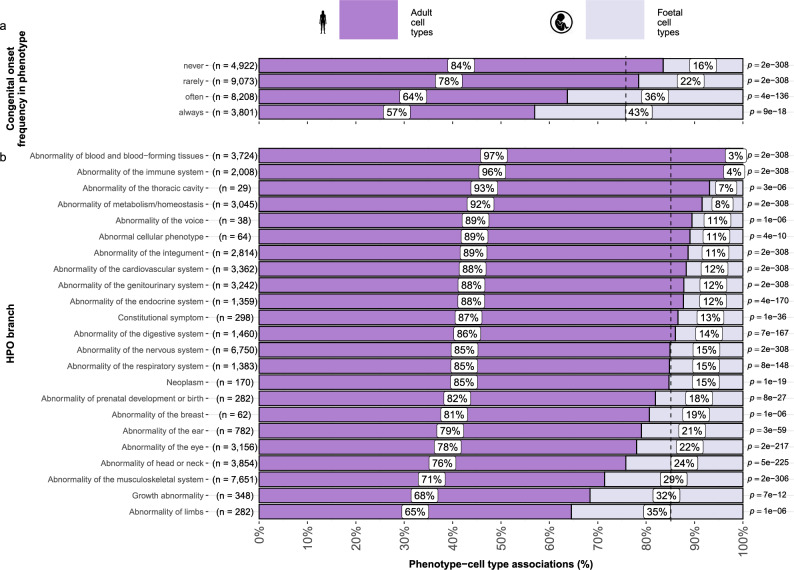
Foetal vs. adult cell type references provide developmental context to phenotype etiology. **a**, Congenital phenotypes are more often associated with foetal cell types. As a phenotype is more often congenital in nature, the greater proportion of foetal cell types are significantly associated with it. **b**, The proportion of phenotype-cell type association tests that are enriched for foetal cell types within each HPO branch. The *p*-values to the right of each bar are the results of an additional series of $\chi ^2$ tests to determine whether the proportion of foetal vs. non-foetal cell types significantly differs from the proportions expected by chance (the dashed vertical line). The foetal silhouette was generated with DALL-E [48]. The adult silhouette is from phylopic.org and is freely available via CC0 1.0 Universal Public Domain Dedication

**Fig. 8:**
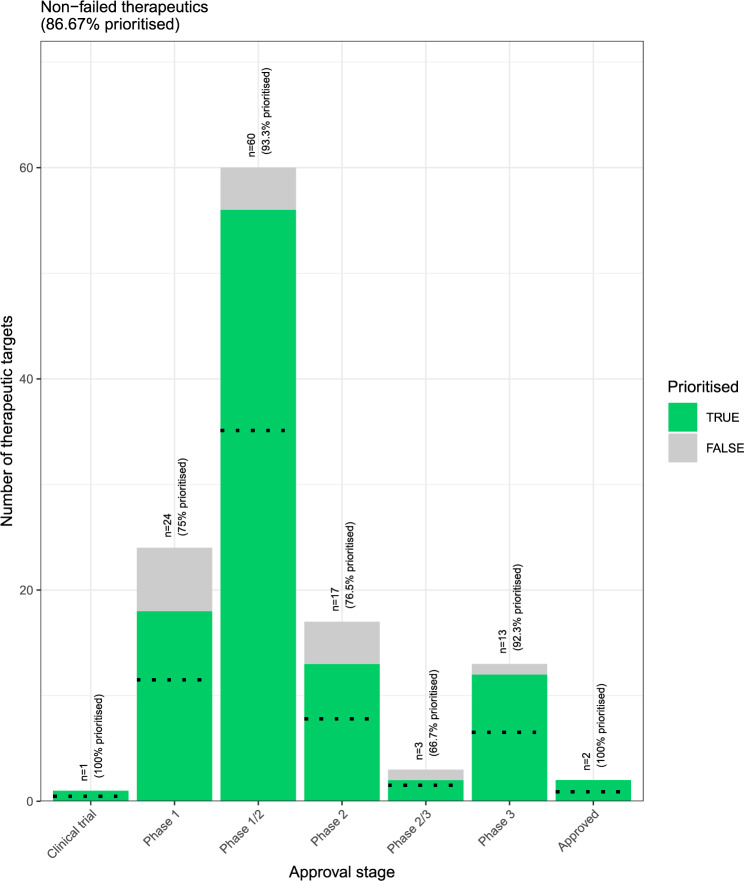
Prioritised targets recapitulate existing gene therapy targets. The proportion of existing gene therapy targets (documented in the Therapeutic Target Database) recapitulated by our prioritisation pipeline. Therapeutics are stratified by the stage of clinical development they were at during the time of writing. While our prioritized targets did not include any failed (‘Terminated’) therapies, the fact that only one such therapy exists in the dataset precludes us from making any conclusions about depletion of failed gene therapy targets in our prioritised targets list. Black dashed lines indicate the number of prioritised targets expected by chance

**Fig. 9:**
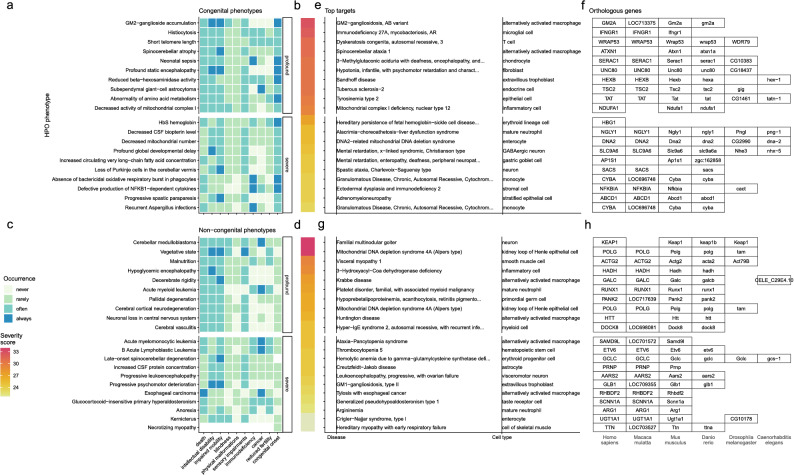
Evidence-based pipeline nominates causal mechanisms to target for gene therapy. Shown here are the top 40 prioritised gene therapy targets at multiple biological scales, stratified by congenital (top row) vs. non-congenital phenotypes (bottom row) as well as severity class (“profound” or “severe”). In this plot, only the top 10 most severe phenotypes within a given strata/substrata are shown **a**, **c**, Severity annotation generated by GPT-4. **b**, **d**, Composite severity scores computed across all severity metrics. **e**, **g**, Top mediator disease and cell type-specific target for each phenotype. **f**, **h** top target gene for each phenotype within humans (*Homo sapiens*). We also include the 1:1 ortholog of each human gene in several commonly used animal models, including monkey (*Macaca mulatta*), mouse (*Mus musculus*), zebrafish (*Danio rerio*), fly (*Drosophila melanogaster*) and nematode (*Caenorhabditis elegans*). Boxes are empty where no 1:1 ortholog is known. See supplement Additional file 1: Fig. S9 for network plots of cell type-specific gene therapy targets for several severe phenotypes and their associated diseases

The remainder of this article describes the pipeline and its outputs at successive levels of resolution: an overview of the study design (Fig. 1), the full set of phenotype-cell type associations (Fig. 2), the relationship between phenotype ontology level and association power (Fig. 3), targeted analyses of recurrent Neisserial infections and their cellular drivers (Figs. 4, 5), severity-stratified cell type findings (Fig. 6), congenital phenotype enrichment for foetal cell types (Fig. 7), and therapeutic target prioritisation with cross-validation against the Therapeutic Target Database (Figs. 8, 9) [23].

## Methods

### Human Phenotype Ontology

The HPO (release 2024-02−08) was downloaded from the EMBL-EBI Ontology Lookup Service [24] and imported into R using the HPOExplorer package. This R object was used to extract ontological relationships between phenotypes as well as to assign absolute and relative ontological levels to each phenotype. The latest version of the HPO phenotype-to-gene mappings and phenotype annotations were downloaded from the official HPO GitHub repository and imported into R using HPOExplorer. This contains lists of genes associated with phenotypes via particular diseases, formatted as three columns in a table (gene, phenotype, disease).

However, not all genes have equally strong evidence of causality with a disease or phenotype, especially when considering that the variety of resources used to generate these annotations (OMIM, Orphanet, DECIPHER) use variable methodologies (e.g. expert-curated review of the medical literature vs. automated text mining of the literature). Because of this, formalizing phenotypes (or any biological entity) as unweighted gene sets can lead to loss of information about the most relevant signals. This is especially true when gene sets become large and poorly supported, essentially decreasing the signal-to-noise ratio [25–27].

Therefore we imported data from the Gene Curation Coalition (GenCC) [28, 29], which (as of 2025-11−30) contains 24,124 evidence scores across 7,566 diseases and 5,533 genes. Evidence scores are defined by GenCC using a standardised ordinal rubric which we then encoded as a semi-quantitative score ranging from 0 (no evidence of disease-gene relationship) to 6 (strongest evidence of disease-gene relationship) (see Additional file 2: Table S5). As each Disease-Gene pair can have multiple entries (from different studies) with different levels of evidence, we then summed evidence scores per Disease-Gene pair to generate aggregated Disease-by-Gene evidence scores. This procedure can be described as follows.

Let us denote:

- $$D$$ as diseases.
- $P$ as phenotypes in the HPO.
- $$G$$ as genes
- $S$ as the evidence scores describing the strength of the relationship between each Disease-Gene pair.
- $M_{ij}$ as the aggregated Disease-by-Gene evidence score matrix.

$$\begin{aligned} M_{ij} = \sum \limits _{k=1}^{\text {f}} D_i G_j S_k \end{aligned}$$

Next, we extracted Disease-Gene-Phenotype relationships from the annotations file distributed by the HPO (*phenotype_to_genes.txt*). This provides a list of genes associated with phenotypes via particular diseases, but does not include any strength of evidence scores.

Here we define:

- $A_{ijk}$ as the Disease-Gene-Phenotype relationships.
- $D_i$ as the $i$th disease.
- $G_j$ as the $j$th gene.
- $P_k$ as the $k$th phenotype.

$$\begin{aligned} A_{ijk} = D_i G_j P_k \end{aligned}$$

In order to assign evidence scores to each Phenotype-Gene relationship, we combined the aforementioned datasets from GenCC ($M_{ij}$) and HPO ($A_{ijk}$) by merging on the gene and disease ID columns. For each phenotype, we then computed the mean of Disease-Gene scores across all diseases for which that phenotype is a symptom. This resulted in a final 2D tensor of Phenotype-by-Gene evidence scores ($L_{ij}$):
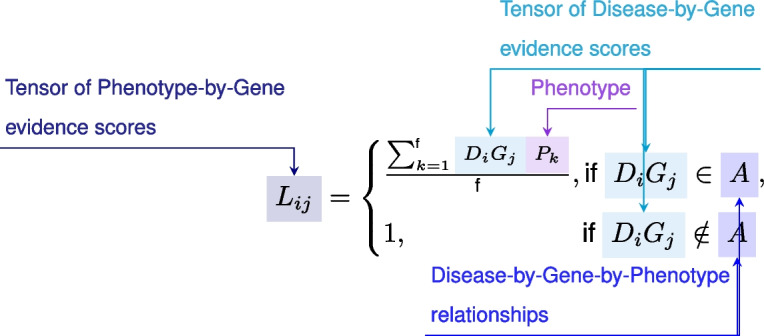


### Construction of the tensor of Phenotype-by-Gene evidence scores

Histograms of evidence score distributions at each step in processing can be found in Additional file 1: Fig. S1.

### Single-cell transcriptomic atlases

In this study, the gene by cell type specificity matrix was constructed using the Descartes Human transcriptome atlas of foetal gene expression, which contains a mixture of single-nucleus and single-cell RNA-seq data (collected with sci-RNA-seq3) [18]. After filtering and down-sampling, this dataset comprised 377,456 cells (from the $\sim$4 million in the full atlas) representing 77 distinct cell types across 15 tissues. All 121 human foetal samples ranged from 72 to 129 days in estimated postconceptual age. To independently replicate our findings, we also used the Human Cell Landscape which contains single-cell transcriptomic data (collected with microwell-seq) from embryonic, foetal, and adult human samples across 49 tissues [19].

Specificity matrices were generated separately for each transcriptomic atlas using the R package EWCE (v1.11.3) [30]. Within each atlas, cell types were defined using the authors’ original freeform annotations in order to preserve the granularity of cell subtypes as well as incorporate expert-identified rare cell types. Cell types were only aligned and aggregated to the level of corresponding Cell Ontology (CL; release v2023-09–21) [31] annotations afterwards when generating summary figures and performing cross-atlas analyses. Using the original gene-by-cell count matrices from each single-cell atlas, we computed gene-by-cell type expression specificity matrices as follows. Genes with very low expression across any cell types were considered to be uninformative and were therefore removed from the input gene-by-cell matrix $F_{gic}$.

Next, we calculated the mean expression per cell type and normalised the resulting matrix to transform it into a gene-by-cell type expression specificity matrix ($S_{gc}$). In other words, each gene in each cell type had a 0–1 score where 1 indicated the gene was mostly specifically expressed in that particular cell type relative to all other cell types. This procedure was repeated separately for each of the single-cell atlases and can be summarised as:
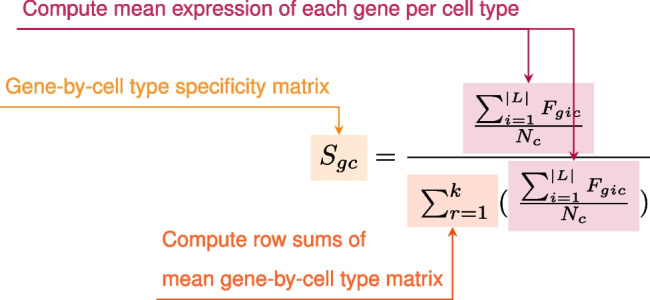


### Phenotype-cell type associations

To test for relationships between each pairwise combination of phenotype ($n=11,047$) and cell type ($n=201$) we ran a series of univariate generalised linear models (GLMs) implemented via the MSTExplorer::run_phenomix function in R (which uses stats::glm internally).

First, we filtered the gene-by-phenotype evidence score matrix ($L_{ij}$) and the gene-by-cell type expression specificity matrix ($S_{gc}$) to only include genes present in both matrices ($n=4,949$ genes in the Descartes Human analyses; $n=4,653$ genes in the Human Cell Landscape analyses). Then, within each matrix any rows or columns with a sum of 0 were removed as these were uninformative data points that did not vary. To improve interpretability of the results $\beta$ coefficient estimates across models (i.e. effect size), we performed a scaling prestep on all dependent and independent variables. Initial tests showed that this had virtually no impact on the total number of significant results or any of the benchmarking metrics based on *p*-value thresholds (Fig. 2). This scaling prestep improved our ability to rank cell types by the strength of their association with a given phenotype as determined by separate linear models.

We repeated the aforementioned procedure separately for each of the single-cell references. Once all results were generated using both cell type references (2,206,994 association tests total), we applied the Benjamini-Hochberg false discovery rate (FDR) [32] (denoted as $FDR_{pc}$) to account for multiple testing. Of note, we applied this correction across all results at once (as opposed to each single-cell reference separately) to ensure the $FDR_{pc}$ was stringently controlled for across all tests performed in this study.

For the significant phenotype-cell type associations, we also wished to identify which genes were most strongly driving this association. We therefore designed a heuristic to consistently extract such *driver genes*. For a given phenotype-cell type pair, *driver genes* were defined as the intersect of genes that had a phenotype evidence score >0 and were within the top 75th expression specificity percentile (quantiles 30–40 out of 40) for the associated cell type. This can be described as follows.

Let

- $G$ be the full set of genes,
- $s_i$ be the phenotype evidence score for gene $i$,
- $e_i$ be the expression specificity of gene $i$ in the associated cell type,
- $q(e_i)$ be the empirical specificity percentile of $e_i$ (ranging from 1 to 40).

We define two subsets:

$$\begin{aligned} A = \{\, i \in G : s_i> 0 \,\}, \end{aligned}$$

$$\begin{aligned} B = \{\, i \in G : q(e_i) \ge 30 \,\}. \end{aligned}$$

The set of driver genes for that phenotype–cell type pair is then

$$\begin{aligned} D = A \cap B. \end{aligned}$$

Framing association testing as a regression problem rather than a gene set enrichment problem has additional benefits, including speed. This approach allowed us to complete all tests within $\sim$30 min on a MacBook Pro with 8 CPUs, while early tests indicated that gene set enrichment-based methods like EWCE [30] would take days to weeks on a high-performance computing cluster to complete the same number of tests due to its computationally expensive bootstrapping procedure. These tests also showed that EWCE was unable to recover nearly as many true positive results (as indicated by our Monarch Knowledge Graph (MKG) benchmark) with a comparable degree of precision (see related Issue for further details: https://github.com/neurogenomics/rare_disease_celltyping/issues/51).

A programmatic implementation of this heuristic can be found in the MSTExplorer::add_driver_genes R function.

### Symptom-cell type associations

In the HPO, a phenotype can be associated with multiple diseases via different subsets of genes (Additional file 1: Fig. S2). Here we define a symptom as a phenotype as it presents within the context of the specific disease. A symptom’s gene set is therefore the subset of genes connecting a phenotype to a specific disease. For example, the phenotype ‘Headache’ (*HP:0002315*) is associated with 346 genes across 192 different diseases. Our association tests described above use evidence scores from all 346 of these genes when testing for a relationship between ‘Headache’ and various cell types. However, the disease ‘Erythrocytosis, familial, 1’ (*OMIM:133100*) is linked to the ‘Headache’ phenotype via a single gene; *EPOR*. Thus, the symptom ‘Headache due to Erythrocytosis, familial, 1’ would have only *EPOR* as its gene list. Another disease, ‘Migraine with or without aura, susceptibility to, 1’ (*OMIM:157300*) is linked with ‘Headache’ via 3 different genes (*ESR1*, *TNF*, *EDNRA*), so the symptom ‘Headache’ in the presence of this disease would consist of these 3 genes.

More formally, the features of a given symptom can be described as the subset of genes annotated to phenotype $p$ via a particular disease $d$, denoted as $G_{dp}$ (see Additional file 1: Fig. S2). We then computed the intersect between symptom genes ($G_{dp}$) and driver genes (described in the previous section, and denoted as $G_{pc}$), resulting in the gene subset $G_{d \cap p \cap c}$. Only $G_{d \cap p \cap c}$ gene sets with 25% or greater overlap with the symptom gene subset ($G_{dp}$) were kept. This procedure was repeated for all phenotype-cell type-disease triads, which can be summarised as follows:
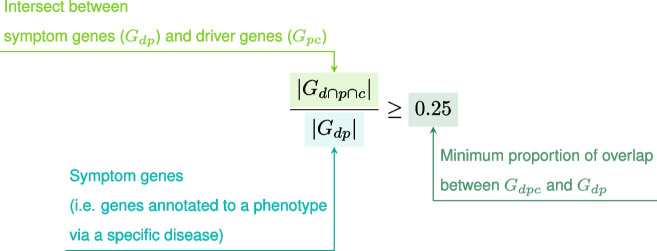


### Validation of expected phenotype-cell type relationships

We first sought to confirm that our tests (across both single-cell references) were able to recover expected phenotype-cell type relationships across seven high-level branches within the HPO (Fig. 2), including abnormalities of the cardiovascular system, endocrine system, eye, immune system, musculoskeletal system, nervous system, and respiratory system. Within each branch the number of significant tests in a given cell type were plotted (Fig. 2b). Mappings between freeform annotations (the level at which we performed our phenotype- cell type association tests) – assigned by the original atlas authors and their closest CL term equivalents, respectively – were provided by CellxGene [33]. CL terms along the *x-axis* of Fig. 2b were assigned colours corresponding to which HPO branch showed the greatest number of enrichments (after normalising within each branch to account for differences in scale). The normalised colouring allows readers to quickly assess which HPO branch was most often associated with each cell type, while accounting for differences in the number of phenotypes across branches. We then ran a series of Analysis of Variance (ANOVA) tests to determine whether (within a given branch) a given cell type was more often enriched (FDR<0.05) within that branch relative to all of the other HPO branches of an equivalent level in the ontology (including all branches not shown in Fig. 2b). After applying Benjamini-Hochberg multiple testing correction [32] (denoted as $FDR_{bc}$), we annotated each respective branch-by-cell type bar according to the significance (****: $FDR_{bc}<1e-04$, ***: $FDR_{bc}<0.001$, **: $FDR_{bc}<0.01$, *: $FDR_{bc}<0.05$). Cell types in Fig. 2a-b were ordered along the *x-axis* according to a dendrogram derived from the CL ontology (Fig. 2c), which provides ground-truth semantic relationships between all cell types (e.g. different neuronal subtypes are grouped together).

As an additional measure of the accuracy of our phenotype-cell types test results we identified conceptually matched branches across the HPO and the CL (Fig. 2d and Additional file 2: Table S6). For example, ‘Abnormality of the cardiovascular system’ in the HPO was matched with ‘cardiocytes’ in the CL which includes all cell types specific to the heart. Analogously, ‘Abnormality of the nervous system’ in the HPO was matched with ‘neural cell’ in the CL which includes all descendant subtypes of neurons and glia. This cross-ontology matching was repeated for each HPO branch and can be referred to as on-target cell types. Within each branch, the $-log_{10}(FDR_{pc})$ values of on-target cell types were binned by rounding to the nearest integer (*x-axis*) and the percentage of tests for on-target cell types relative to all cell types were computed at each bin (*y-axis*) (Fig. 2d). The baseline level (dotted horizontal line) illustrates the percentage of on-target cell types relative to the total number of observed cell types. Any percentages above this baseline level represent greater than chance representation of the on-target cell types in the significant tests.

### Validation of inter- and intra-dataset consistency

We tested for inter-dataset consistency of our phenotype-cell type association results across different single-cell reference datasets (Descartes Human and Human Cell Landscape). For association tests with exactly matching Cell Ontology ID across the two references, we tested for a relationship between the effect sizes (each GLM’s $R^2$ estimates) generated with each of the references using Pearson correlation (stats::cor.test(method="pearson")). We repeated this correlation analysis using only the phenotype-cell type associations that were significant (FDR < 0.05) in both reference datasets.

We also tested for intra-dataset consistency within the Human Cell Landscape by running additional correlations between the phenotype-cell type association test statistics of the foetal and the adult samples (again using estimates from all results, and significant-only results at FDR < 0.05).

To confirm the correlation findings, we repeated these analyses using a permutation procedure (1,000 permutations) in which we generate a null distribution by randomly shuffling the IC values across phenotypes. We then derive a two-sided empirical *p*-value by calculating the proportion of the null distribution’s correlation coefficient that were greater than or equal to the observed correlation coefficient. This procedure is implemented in the function MSTExplorer::run_permutation_test_cor.

Finally, we compute the symmetric replication rate within each of the comparison described above (between scRNA-seq references, and between developmental stages within the Human Cell Landscape). This was defined as the proportion of significant results (FDR < 0.05) in dataset A that were also significant (FDR < 0.05) in dataset B, and vice versa, averaged across both directions. This can be described as follows.

**Variable descriptions**

- $S_{A}$: The set of phenotype–cell-type associations that pass the significance threshold (e.g., $FDR < 0.05$) in dataset $A$.
- $S_{B}$: The set of phenotype–cell-type associations that pass the same significance threshold in dataset $B$.
- $S_{A} \cap S_{B}$: The set of associations that are significant in *both* datasets.
- $$RR$$: Replication rate.

**Directional replication rate (A** $\rightarrow$ **B)**

$$\begin{aligned} RR_{A \rightarrow B} = \frac{ \left| S_{A} \cap S_{B} \right| }{ \left| S_{A} \right| } \end{aligned}$$

**Directional replication rate (B** $\rightarrow$ **A)**

$$\begin{aligned} RR_{B \rightarrow A} = \frac{ \left| S_{A} \cap S_{B} \right| }{ \left| S_{B} \right| } \end{aligned}$$

**Symmetric replication rate**

$$\begin{aligned} RR_{sym} = \frac{ \left( RR_{A \rightarrow B} + RR_{B \rightarrow A} \right) }{2} \end{aligned}$$

### More specific phenotypes are associated with fewer genes and cell types

To explore the relationship between HPO phenotype specificity and various metrics from our results, we computed the information content (IC) [34] scores for each term in the HPO. IC is a measure of how much specific information a term within an ontology contains. In general, terms deeper in an ontology (closer to the leaves) are more specific, and thus informative, than terms at the very root of the ontology (e.g. ‘Phenotypic abnormality’). Where $k$ denotes the number of offspring terms (including the term itself) and $N$ denotes the total number of terms in the ontology, IC can be calculated as:

$$\begin{aligned} IC=-log\left( \frac{k}{N}\right) \end{aligned}$$

Next, IC scores were quantised into 10 bins using the ceiling R function to improve visualisation. We then performed a series of linear regressions between phenotype binned IC scores and: 1) number of genes annotated per HPO phenotype, 2) the number of significantly associated cell types per HPO phenotype, and 3) the model estimate of each significant phenotype-cell type associations (at FDR < 0.05) after taking the log of the absolute value ($log_2(|estimate|)$).

### Monarch Knowledge Graph recall

Finally, we gathered known phenotype-cell type relationships from the MKG, a comprehensive database of links between many aspects of disease biology [35]. This currently includes 103 links between HPO phenotypes ($n=103$) and CL cell types ($n=79$). Of these, we only considered the 82 phenotypes that we were able to test given that our ability to generate associations was dependent on the existence of gene annotations within the HPO. We considered instances where we found a significant relationship between exactly matching pairs of HPO-CL terms as a hit.

However, as the cell types in MKG were not necessarily annotated at the same level as our single-cell references, we considered instances where the MKG cell type was an ancestor term of our cell type (e.g. ‘myeloid cell’ vs. ‘monocyte’), or *vice versa*, as hits. We also adjusted ontological distance by computing the ratio between the observed ontological distance and the smallest possible ontological distance for that cell type given the cell types that were available in our references ($dist_{adjusted}=(\frac{dist_{observed}+1}{dist_{minimum}+1})-1$). This provides a way of accurately measuring how dissimilar our identified cell types were for each phenotype-cell type association (Additional file 1: Fig. S4).

### Prioritising phenotypes based on severity

Only a small fraction of the phenotypes in HPO (<1%) have metadata annotations containing information on their time course, consequences, and severity. This is due to the time-consuming nature of manually annotating thousands of phenotypes. To generate such annotations at scale, we previously used Generative Pre-trained Transformer 4 (GPT-4), a large language model (LLM) as implemented within OpenAI’s Application Programming Interface (API) [22]. After extensive prompt engineering and ground-truth benchmarking, we were able to acquire annotations on how often each phenotype directly causes intellectual disability, death, impaired mobility, physical malformations, blindness, sensory impairments, immunodeficiency, cancer, reduced fertility, or is associated with a congenital onset. These criteria were previously defined in surveys of medical experts as a means of systematically assessing phenotype severity [36]. Responses for each metric were provided in a consistent one-word format which could be one of: ‘never’, ‘rarely’, ‘often’, ‘always’. This procedure was repeated in batches (to avoid exceeding token limits) until annotations were gathered for 16,982/18,082 HPO phenotypes.

We then encoded these responses into a semi-quantitative scoring system (‘never’=0, ‘rarely’=1, ‘often’=2, ‘always’=3), which were then weighted by multiplying a semi-subjective scoring of the relevance of each metric to the concept of severity on a scale from 1–6, with 6 being the most severe (‘death’=6, ‘intellectual_disability’=5, ‘impaired_mobility’=4, ‘physical_malformations’=3, ‘blindness’=4, ‘sensory_impairments’=3, ‘immunodeficiency’=3, ‘cancer’=3, ‘reduced_fertility’=1, ‘congenital_onset’=1). Finally, the product of the score was normalised to a quantitative severity score ranging from 0–100, where 100 is the theoretical maximum severity score. This phenotype severity scoring procedure can be expressed as follows.

Let us denote:

- $p$: a phenotype in the HPO.
- $j$: the identity of a given annotation metric (i.e. clinical characteristic, such as ‘intellectual disability’ or ‘congenital onset’).
- $W_j$: the assigned weight of metric $j$.
- $F_j$: the maximum possible value for metric $j$, equal to 3 (“always”). This value is equivalent across all $j$ annotations.
- $F_{pj}$: the numerically encoded value of annotation metric $j$ for phenotype $p$.
- $NSS_p$: the final composite severity score for phenotype $p$ after applying normalisation to align values to a 0–100 scale and ensure equivalent meaning regardless of which other phenotypes are being analysed in addition to $p$. This allows for direct comparability of severity scores across studies with different sets of phenotypes.

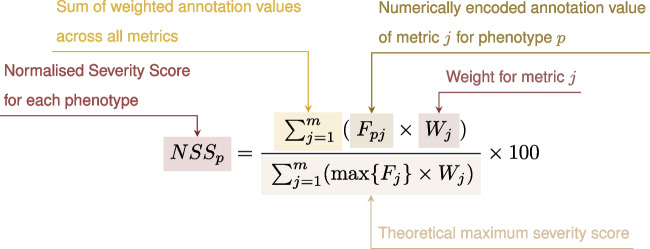


Using the numerically encoded GPT annotations (0=“never”, 1=“rarely”, 2=“often”, 3=“always”) we computed the mean encoded value per cell type within each annotation. One-sided Wilcoxon rank-sum tests were run using the rstatix::wilcox_test() function to test whether each cell type was associated with more severe phenotypes relative to all other cell types. This procedure was repeated for severity annotation independently (death, intellectual disability, impaired mobility, etc.) Fig. 6a. Next, we performed a Pearson correlation test between the number of phenotypes that a cell type is significantly associated with (at FDR<0.05) has a relationship with the mean composite GPT severity score of those phenotypes (Fig. 6b). This was performed using the ggstatsplot::ggscatterstats() R function.

### Congenital phenotypes are associated with foetal cell types

The GPT-4 annotations also enabled us to assess whether foetal cell types were more often significantly associated with congenital phenotypes in our Human Cell Landscape results as this single-cell reference contained both adult and foetal versions of cell types (Fig. 7). To do this, we performed a chi-squared ($\chi ^2$) test on the proportion of significantly associated cell types containing any of the substrings or (within cell types annotations from the original Human Cell Landscape authors [19]) vs. those associated without, stratified by how often the corresponding phenotype had a congenital onset according to the GPT phenotype annotations (including ‘never’, ‘rarely’, ‘often’, ‘always’). In addition, a series of $\chi ^2$ tests were performed within each congenital onset frequency strata, to determine whether the observed proportion of foetal cell types vs. non-foetal cell types significantly deviated from the proportions expected by chance.

We next tested whether the proportion of tests with significant associations with foetal cell types varied across the major HPO branches using a $\chi ^2$ test. We also performed separate $\chi ^2$ test within each branch to determine whether the proportion of significant associations with foetal cell types was significantly different from chance.

Next, we aimed to create a continuous metric from −1 to 1 that indicated how biased each phenotype is towards associations with the foetal or adult form of a cell type. For each phenotype we calculated the foetal-adult bias score as the difference in the association *p*-values between the foetal and adult version of the equivalent cell type ($\text {foetal-adult bias}: p_{adult} - p_{foetal} = \Delta p \in [-1,1]$). A score of 1 indicates the phenotype is only associated with the foetal version of the cell type and −1 indicates the phenotype is only associated with the adult version of the cell type.

In order to summarise higher-order HPO phenotype categories that were most biased towards foetal or adult cell types, ontological enrichment tests were run on the phenotypes with the top/bottom 50 greatest/smallest foetal-adult bias scores. The enrichment tests were performed using the simona::dag_enrich_on_offsprings function, which uses a hypergeometric test to determine whether a list of terms in an ontology are enriched for offspring terms (descendants) of a given ancestor term within the ontology. Phenotype categories with an HPO ontological enrichment *p*-value < 0.05 were considered significant.

We were similarly interested in which higher-order cell type categories tended to be most commonly associated with these strongly foetal-/adult-biased phenotypes. Another set of ontological enrichment tests were run on the cell types associated with the top/bottom 50 phenotypes from the previous analysis. The CL ontology-aligned IDs for each group of cell types were fed into the simona::dag_enrich_on_offsprings using the CL ontology. Significantly enriched cell type categories were defined as those with a CL ontological enrichment *p*-value < 0.05.

### Therapeutic target identification

We developed a systematic and automated strategy for identifying putative cell type-specific gene targets for each phenotype based on a series of filters at phenotype, cell type, and gene levels.

First, we transformed our phenotype-cell type association results and merged them with primary data sources (e.g. GenCC gene-disease relationships, scRNA-seq atlas datasets) to create a large table of multi-scale relationships, where each row represented a tetrad of disease-phenotype-cell type-gene relationships. We then filtered non-significant phenotype-cell type relationships (only associations with $FDR<0.05$) as well as phenotype-gene relationships with strong causal evidence ($\text {GenCC score}>3$). We also removed any phenotypes that were too broad to be clinically useful, as quantified using the information content (IC) ($IC>8$), which measures the how specific each term is within an ontology (i.e. HPO). Gene-cell type relationships were established by taking genes that had the top 25% expression specificity quantiles within each cell type. When connecting cell types to diseases via phenotypes, we used a symptom intersection threshold of >0.25. Next, we sorted the remaining results in descending order of phenotype severity using the GPT-4 composite severity scores described earlier. Finally, to limit the size of the resulting multi-scale networks we took only the top 10 rows, where each row represented a tetrad of disease-phenotype-cell type-gene relationships. This resulted in number of relatively small, high-confidence disease-phenotype-cell type-gene networks that could be reasonably interrogated through manual inspection and network visualisation. For example, if one was interested in the mechanisms causing ‘Recurrent Neisserial infections’, one would need only select all rows that include this phenotype to find all of its most relevant connection to diseases, cell types, and genes.

The entire target prioritisation procedure can be replicated with a single function: MSTExplorer::prioritise_targets. This function automates all of the reference data gathering (e.g. phenotype metadata, cell type metadata, cell type signature reference, gene lengths, severity tiers) and takes a variety of arguments at each step for greater customisability. Each step is described in detail in Additional file 2: Table S3. Phenotypes that often or always caused physical malformations (according to the GPT-4 annotations) were also removed from the final prioritised targets list, as these were unlikely to be amenable to gene therapy interventions. Finally, phenotypes were sorted by their composite severity scores such that the most severe phenotypes were ranked the highest.

### Therapeutic target validation

To assess whether our prioritised therapeutic targets were likely to be viable, we computed the overlap between our gene targets and those of existing gene therapies at various stages of clinical development (Fig. 8). Gene targets were obtained for each therapy from the Therapeutic Target Database (TTD; release 2026-06−02) [23] and mapped onto standardised HUGO Gene Nomenclature Committee (HGNC) gene symbols using the orthogene R package. We stratified our overlap metrics according to whether the therapies had failed (unsuccessful clinical trials or withdrawn), or were non-failed (successful or ongoing clinical trials). We then conducted hypergeometric tests to determine whether the observed overlap between our prioritised targets and the non-failed therapy targets was significantly greater than expected by chance (i.e. enrichment). We also conducted a second hypergeometric test to determine whether the observed overlap between our prioritised targets and the failed therapy targets was significantly less than expected by chance (i.e. depletion). Finally, we repeated the analysis against all therapeutic targets, not just those of gene therapies, to determine whether our prioritised targets had relevance to other therapeutic modalities.

### Experimental model translatability

To improve the likelihood of successful translation between preclinical animal models and human patients, we created an interspecies translatability prediction tool for each phenotype nominated by our gene therapy prioritised pipeline (Additional file 1: Fig. S8). First, we extracted ontological similarity scores of homologous phenotypes across species from the MKG [35]. Briefly, the ontological similarity scores ($SIM_o$) are computed for each homologous pair of phenotypes across two ontologies by calculating the overlap in homologous phenotypes that are ancestors or descendants of the target phenotype. Next, we generated genotypic similarity scores ($SIM_g$) for each homologous phenotype pair by computing the proportion of 1:1 orthologous genes using gene annotation from their respective ontologies. Interspecies orthologs were also obtained from the MKG. Finally, both scores are multiplied together to yield a unified ontological-genotypic similarity score ($SIM_{og}$).

### Novel R packages

To facilitate all analyses described in this study and to make them more easily reproducible by others, we created several open-source R packages. KGExplorer (v0.99.10) imports and analyses large-scale biomedical knowledge graphs and ontologies. HPOExplorer (v1.0.6) aids in managing and querying the directed acyclic ontology graph within the HPO. MSTExplorer (v1.0.10) facilitates the efficient analysis of many thousands of phenotype-cell type association tests, and provides a suite of multi-scale therapeutic target prioritisation and visualisation functions. These R packages also include various functions for distributing the post-processed results from this study in an organised, tabular format. Of note, MSTExplorer::load_example_results loads all summary statistics from our phenotype-cell type tests performed here.

### Computing environment

All analyses were performed in R version 4.5.1 (2025-06−13). Key R package dependencies and their versions were: data.table (v1.18.2.1), ggplot2 (v4.0.2), simona (v1.8.1) for ontology operations, orthogene (v1.17.3) for cross-species gene mapping, and Seurat (v5.4.0) for single-cell data handling. The manuscript itself was prepared with Quarto and rendered to PDF via LuaLaTeX (TeX Live 2025).

### Rare Disease Celltyping Portal

To further increase the ease of access for stakeholders in the RD community without the need for programmatic experience, we developed a series of web apps to interactively explore, visualise, and download the results from our study. Collectively, these web apps are called the Rare Disease Celltyping Portal. The website can be accessed at https://neurogenomics-ukdri.dsi.ic.ac.uk/.

The Rare Disease Celltyping Portal integrates diverse datasets, including the HPO, cell types, genes, and phenotype severity, into a unified platform that allows users to perform flexible, bidirectional queries. Users can start from any entry point: either phenotype, cell type, genes, or severity, and seamlessly trace relationships across these dimensions.

The portal provides a dynamic and intuitive exploration experience with its real-time interaction capabilities and responsive interface including network graphs, bar charts, and heat maps. It has the ability to handle large datasets efficiently and offer fast query response by building with FARM stack (FastAPI, React, MongoDB). The portal is designed for a broad audience, including researchers, clinicians, and biologists, by offering user-friendly navigation and interactive visual outputs. By enabling users to intuitively explore complex biological relationships, the portal aims to accelerate rare disease research, enhance diagnostic accuracy, and drive therapeutic innovation.

All code used to generate the website can be found at https://github.com/neurogenomics/Rare-Disease-Web-Portal.

### Mappings

Mappings from the HPO to other medical ontologies were extracted from the EMBL-EBI Ontology Xref Service (OxO; https://www.ebi.ac.uk/spot/oxo/) by selecting the National Cancer Institute metathesaurus (NCIm) as the target ontology and either “SNOMED CT”, “UMLS”, “ICD-9” or “ICD-10 CM” as the data source. HPO terms were then selected as the ID framework with to mediate the cross-ontology mappings. Mappings between each pair of ontologies were then downloaded, stored in a tabular format. The mappings files can be accessed with the function HPOExplorer::get_mappings or directly via the HPOExplorer Releases page on GitHub (https://github.com/neurogenomics/HPOExplorer/releases/tag/latest).

## Results

### Phenotype-cell type associations

We systematically investigated cell types underlying HPO phenotypes, hypothesising that genes with cell type–specific expression are most relevant to those cell types, and that disrupting such genes will have variable effects across cellular contexts. More precisely, for each phenotype we created a list of associated genes weighted by the strength of the evidence supporting those associations, imported from the Gene Curation Coalition (GenCC) [28]. Analogously, we created mean gene expression profiles for each cell type using scRNA-seq atlases and then normalised them to compute cell type specificity of gene expression (see Methods subsection *Single-cell transcriptomic atlases* for further details).

For comprehensiveness, we used two pan-tissue scRNA-seq atlases: Descartes Human ($\sim$4 million single-nuclei and single-cells from 15 foetal tissues) [18] and Human Cell Landscape ($\sim$703,000 single-cells from 49 embryonic, foetal and adult tissues) [19]. For every unique combination of phenotype and cell type, we trained a generalised linear regression model to test for association between the respective gene-phenotype association scores and gene-cell type expression specificity scores (Fig. 1). We then applied stringent multiple testing correction to control the FDR across all tests, and significant phenotype–cell type associations were identified at FDR<0.05.

In Descartes Human, 19,929/848,078 (2.35%) tests were significant across 77 cell types and 7,340 phenotypes. In Human Cell Landscape, the corresponding values were 1.96% significant tests, 124 cell types, and 9,049 phenotypes, with more phenotypes linked to at least one cell type due to greater cell type diversity and life-stage coverage. Across both atlases, the median number of significant cell types per phenotype was 3, indicating specificity of associations. Overall, 8,628/8,631 (>99.96%) of diseases had significant cell type associations for at least one phenotype. Full stratified results are provided in Additional file 2: Table S2.

### Validation of expected phenotype-cell type relationships

We intuit that organ system-specific abnormalities are often driven by cell types within that system. The HPO’s high-level categories allow systematic testing; for example, heart phenotypes should typically involve cardiocytes, and nervous system abnormalities should involve neural cells. All cell types in our single-cell atlases were mapped to the Cell Ontology (CL), a hierarchical vocabulary of cell types.

A cell type was considered *on-target* for an HPO branch if it belonged to a matching CL branch (Additional file 2: Table S4). For each HPO branch (Fig. 2b), we tested whether cell types were more often associated with phenotypes in that branch compared to all others, and identified those overrepresented at FDR<0.05. All 7 HPO branches showed disproportionate associations with on-target cell types from their respective organ systems.

We hypothesised that more strongly significant phenotype–cell type associations are more likely to be on-target. Grouping $-log_{10}(\text {p-values})$ into six bins, we calculated the proportion of on-target cell types per HPO–CL branch pairing. Indeed, this proportion consistently increased with association significance ($r_{Pearson}=$ $0.63$, $p=$ $1.1 \times 10^{-6}$). For example, in nervous system abnormalities neural cells constituted only $23$% of all tested cell types, yet made up $46$% of associations within the $-log_{10}(\text {p-values}) \ge 6$ bin (which corresponds to $p\le 10^{-7}$). This confirms that stronger associations are more likely to involve on-target cell types, confirming our association strategy captures real relationships.

### Validation of inter- and intra-dataset consistency

If our methodology works, it should yield consistent phenotype-cell type associations across different datasets. We therefore tested for the consistency of our results across the two single-cell reference datasets (Descartes Human vs. Human Cell Landscape) for the subset of overlapping cell types Additional file 1: Fig. S3. In total there were 142,285 phenotype-cell type associations to compare across the two datasets (across 10,945 phenotypes and 13 cell types annotated to the exact same CL term. We found that the correlation between association effect sizes across the two datasets was significant ($r_{Pearson}$=$0.58$, $p$=$1.4 \times 10^{-95}$). Within the subset of phenotype-cell type associations that were significant in both single-cell datasets (FDR<0.05), we found that degree of correlation between the association effect sizes across datasets was even stronger ($r_{Pearson}=$ $0.73$, $p=$ $1.4 \times 10^{-95}$). The symmetric replication rate, i.e. the mean percentage of associations that were significant in both datasets at FDR<0.05, was 39%.

While cell types change their transcriptional signatures over the course of development, we would nevertheless expect there to be some degree of correlation between the developing and mature versions of the same cell types. Therefore, we also checked for the intra-dataset consistency between the *p*-values of the foetal and adult samples in the Human Cell Landscape, showing a very similar degree of correlation as the inter-dataset comparison for full results estimates ($r_{Pearson}=$ $0.44$, $p=$ $2.2 \times 10^{-308}$) and the significant subset ($r_{Pearson}=$ $0.40$, $p=$ $4.5 \times 10^{-92}$). The symmetric replication rate was 36%.

All inter- and intra-dataset correlation tests were confirmed with permutation testing (1,000 permutations, Additional file 2: Table S8). Together, these results suggest that our approach to identifying phenotype-cell type associations is both replicable and generalisable to new datasets.

### More specific phenotypes are associated with fewer genes and cell types

Higher levels of the ontology represent broad classes of phenotype (e.g. ‘Abnormality of the nervous system’) while the lower levels can get very detailed (e.g. ‘Spinocerebellar atrophy’). The higher level phenotypes inherit all genes associated with lower level phenotypes, so naturally they have more genes than the lower level phenotypes (Fig. 3a; $r_{Pearson}=$ $-0.58$, $p<$ $2.2 \times 10^{-308}$).

Next, we reasoned that the more detailed and specific a phenotype is, the more likely it is to be driven by fewer cell types. For example, while ‘Neurodevelopmental abnormality’ could plausibly be driven by any/all cell types in the brain, it is more likely that ‘Impaired visuospatial constructive cognition’ is driven by a select subset of cell types. This was indeed the case, as we observed a strongly significant negative correlation between phenotype level and the number of significantly associated cell types (Fig. 3b; $r_{Pearson}=$ $-0.61$, $p<$ $6.9 \times 10^{-62}$). We also found that the phenotype-cell type association effect size increased with greater phenotype specificity, showing that more specific phenotypes are strongly associated with a refined subset of cell types (Fig. 3c; $r_{Pearson}=$ $0.21$, $p<$ $2.2 \times 10^{-308}$). In all cases, we confirmed the robustness of these results by iteratively comparing the observed correlations against permuted baseline correlations (1,000 permutations, $p<$ $2.2 \times 10^{-308}$; Additional file 2: Table S7).

### Validation of phenotype-cell type associations using biomedical knowledge graphs

To validate phenotype–cell type associations without literature bias, we used the MKG, a curated database of biomedical concepts and relationships containing 103 known associations [35].

The MKG served as a benchmark for the field’s current knowledge. For each MKG association, we calculated the proportion of cell types recovered in our results at different ontological distances in the Cell Ontology. Distance 0 indicates the closest possible match (e.g. “monocyte” vs. “monocyte”), with greater distances reflecting progressively broader matches (e.g. distance 1: “monocyte” vs. “classical monocyte”). The theoretical maximum recall was capped by the percentage of MKG phenotypes for which we identified at least one significant association ($FDR_{pc}$). In other words, since our results only contain significant cell type associations for 90% of the phenotypes in the MKG’s phenotype-cell type associations, our maximum achievable performance was 90% recall.

Our results included at least one significant cell type for $90$% of MKG phenotypes. At distance 0, we recalled $57$% of associations; at distance 1, recall rose to $77$%, reaching a maximum of $90$% at the largest allowed distance. Precision could not be computed, as MKG lists only true positives. Overall, these benchmarks show that our approach recovers most known phenotype–cell type associations while generating many novel ones.

### Phenome-wide analyses discover novel phenotype-cell type associations

Having confirmed many phenotype-cell type associations match prior expectations, we explored novel links for undercharacterised phenotypes. ‘Recurrent bacterial infections’ ($19$ descendants, e.g. staphylococcal, streptococcal, Neisserial) mostly associated with immune cells (e.g. macrophages, dendritic cells, T cells, monocytes, neutrophils) (Fig. 4). Known links include ‘Recurrent staphylococcal infections’ with myeloid cells [37–40], where monocytes were most strongly associated (FDR=$1.0 \times 10^{-30}$, $\beta$=$0.18$). Notably, amongst the recurrent bacterial infection phenotypes, hepatoblasts were exclusively associated with ‘Recurrent Neisserial infections’, hereafter RNI (Descartes Human: FDR=$1.1 \times 10^{-6}$, $\beta$=$8.2 \times 10^{-2}$).

To better understand the multi-scale mechanisms underlying RNI susceptibility, we visualised the putative causal relationships between genes, cell types and diseases associated with RNI as a network (Fig. 5). The phenotype RNI was connected to cell types through the aforementioned association test results (FDR<0.05). Genes that were primarily driving these associations (i.e. genes both strongly linked with RNI and highly specifically expressed in the given cell type) were designated as *driver genes* and retained for plotting (see Methods for details). While a gene being non-specifically expressed does not necessarily mean it is unimportant for the function of a cell (many ubiquitously expressed genes are essential for cell function), gene expression specificity is nevertheless a useful metric to unambiguously link cell types to other biological entities (e.g. phenotypes). Diseases were then connected to phenotypes and gene nodes based on HPO annotations. Finally, diseases were also connected to cell types as a transitive property of being connected to phenotypes, but only if the disease gene set overlapped with 25% or more of the driver genes for that particular phenotype-cell type relationship (see Methods subsection *Symptom-cell type associations* and Additional file 1: Fig. S2 for further explanation).

Using this network-based approach, we found that RNI (a phenotype of 7 diseases: ‘C5 deficiency’, ‘C6 deficiency’, ‘C7 deficiency’, ‘Complement component 8 deficiency, type II’, ‘Complement factor B deficiency’, ‘Complement factor I deficiency’, ‘Mannose-Binding lectin deficiency’) was also linked to stromal cells (FDR=$4.6 \times 10^{-6}$, $\beta$=$7.9 \times 10^{-2}$), stratified epithelial cells (FDR=$1.7 \times 10^{-23}$, $\beta$=$0.15$), and embryonic stem cells (FDR=$5.4 \times 10^{-5}$, $\beta$=$7.4 \times 10^{-2}$). All of the gene implicated in this causal network are part of the complement system (*C5*, *C6*, *C8B*, *CFB*, *CFI*, *MBL2*, and *C7*). Complement deficiencies are known to cause a marked susceptibility to infection [41, 42]. However, the complement system comprises more than 56 genes and can be expressed in a wide variety of cell types [43]. Our analysis was able to decompose the multiple mechanisms underlying RNI into subsets of complement proteins and identify the specific cell types they each affect. For example, disruptions in complement genes *C5*, *C8*, and *C7* cause RNI via hepatoblasts, stratified epithelial cells, and stromal cells, respectively (Fig. 5). These four cell types may represent disease subtypes with distinct clinical courses or biomarkers, allowing us to begin resolving RNI-related disease mechanisms at cell-type resolution.

In particular, we were intrigued by the finding that RNI was associated with hepatoblasts but not their mature counterpart, hepatocytes. Mature hepatocytes are well recognised as the principal source of complement proteins [45]. However, emerging evidence indicates that foetal hepatoblasts also express detectable amounts of complement genes [46, 47], which we confirmed by querying the CellxGene browser [33] (see Data Availability). Upon further inspection, we found that these were in fact circulating hepatoblast-like cells found in liver, placenta and spleen, according to the authors of the Descartes scRNA-seq atlas [18]. These cells express high levels of alpha fetoprotein (AFP), serum albumin (ALB), and apolipoproteins (including APOE). Our results support this hypothesis as these AFP+/ALB+ cells were significantly associated with 12 liver-related phenotypes, as well as 58 blood-related phenotypes. Together, these findings suggest that these hepatoblast-like cell subpopulations play a causal role in complement-related disorders during development. However, this relatively novel cell type must be better characterised before drawing any firm conclusions.

### Prioritising phenotypes based on severity

Some phenotypes are more severe than others and thus could be prioritised for treatment (e.g. ‘Leukonychia’ is far less severe than ‘Leukodystrophy’). To systematically rank phenotypes, we used GPT-4 to annotate severity for 16,982/18,082 ($94$%) HPO phenotypes [22]. Benchmarking against ground-truth HPO branch annotations showed high accuracy (recall=$96$%, min=$89$%, max=$100$%, SD=$4.5$%) and strong consistency ($91$%). From these, we computed weighted severity scores (0–100) for all phenotypes. The most severe was ‘Atrophy/Degeneration affecting the central nervous system’ (*HP:0007367*, score=$47$), followed by ‘Anencephaly’ (*HP:0002323*, score=$45$). There were 677 phenotypes with score 0 (e.g. ‘Thin toenail’), mean=$10$ (median=$9.4$).

Merging severity scores with significant (FDR<0.05) phenotype–cell type associations revealed that neuronal brush cells had the highest average severity, followed by Mueller cells and glial cells, while megakaryocytes had the lowest. Numerically encoding GPT annotations (0–3) and applying Wilcoxon tests confirmed expected links, e.g. retinal pigment epithelial cells with blindness, ventricular cardiac muscle cells with death, and analogous patterns for reduced fertility, immunodeficiency, impaired mobility, and cancer. Finally, we found that cell types associated with more phenotypes also tended to have higher mean composite severity ($p=$ $7.9 \times 10^{-4}$, $\hat{\rho }_{Spearman}$=$0.33$), supporting the idea that broadly involved cell types perform critical physiological functions whose disruption causes more severe disease (Fig. 6).

### Congenital phenotypes are associated with foetal cell types

The life stage at which a phenotype manifests affects treatment options, as some interventions (e.g. gene therapies) may be ineffective once developmental defects occur. In the DescartesHuman dataset all cells were foetal, while the Human Cell Landscape included both embryonic/foetal ($29$% of cell types), and adult tissues ($71$% of cell types). Some cell types exist in both stages (e.g. chondrocytes), while others are foetal-specific (e.g. neural crest cells). Congenital phenotypes (according to our severity annotations) were strongly associated with foetal cell types ($p=$ $4.7 \times 10^{-261}$, $\chi ^2=$ $1.2 \times 10^{3}$), consistent with their developmental origins (Fig. 7a).

HPO branches varied significantly in the proportion of their significant associations with foetal cell types ($\hat{V}_{Cramer}$=$0.22$, $p<$ $2.2 \times 10^{-308}$, Fig. 7b). Branches with the most disproportionate number of foetal cell type associations were ‘Abnormality of limbs’ ($35$%), ‘Growth abnormality’ ($32$%), and ‘Abnormality of the musculoskeletal system’ ($29$%). The most adult-biased branches were ‘Abnormality of blood and blood-forming tissues’ ($97$%) and ‘Abnormality of the immune system’ ($96$%).

Some phenotypes involve only foetal or only adult versions of a cell type. We quantified bias by comparing association *p*-values between foetal and adult versions of the same type (metric range: 1=foetal-only, −1=adult-only). The top $50$ foetal-biased phenotypes revealed were enriched for the HPO branches ‘Abnormal nasal morphology’ ($p=$ $2.4 \times 10^{-7}$) and ‘Abnormal external nose morphology’ ($p=$ $2.5 \times 10^{-6}$), which included specific phenotypes such as ‘Abnormal morphology of the nasal alae’ (see Additional file 2: Table S9, and Additional file 2: Table S10). Adult-biased phenotypes were instead enriched for the branches ‘Abnormal elasticity of skin’ ($p=$ $3.6 \times 10^{-7}$) and ‘Abnormality of the cardiovascular system’ ($p=$ $4.7 \times 10^{-3}$), with examples like ‘Excessive wrinkled skin’ and ‘Paroxysmal supraventricular tachycardia’. These align with known developmental and age-related processes, supporting our approach for linking phenotypes to causal cell types.

### Therapeutic target identification

In the above sections, we demonstrated how gene association databases can be used to investigate the cell types underlying disease phenotypes at scale. While these associations are informative on their own, we wished to take these results further in order to have a more translational impact. Knowledge of the causal cell types underlying each phenotype can be highly informative for scientists and clinicians in their quest to study and treat them. Therapeutic targets with supportive genetic evidence have 2.6x higher success rates in clinical trials [49–51]. Furthermore, knowing which cell types to target with gene therapy can maximise the efficacy of highly expensive payloads, and minimise side effects (e.g. immune reaction to viral vectors). Recent biotechnological advances have greatly enhanced our ability to target specific cell types with gene therapy, making specific and accurate knowledge the correct underlying cell types more pertinent than ever [20, 21].

Rather than consider phenotypes in isolation, or even phenotype associations with particular cell types, we sought to identify multi-scale therapeutic targets. That is, specific genes to target in specific cell types in specific phenotypes as they present in particular diseases. The confluence of these pieces of information is crucial for clinical utility, as it provides the much-needed context to develop effective therapeutics for real-world patient populations. For example, the same phenotype may be caused by disruptions to one of several cell types, each of which are in turn caused by mutations to particular genes. Networks are a natural way to visualize the complex relationships between these various biological entities, and using sensible filters (i.e. pruning) keeps the networks small enough to gain meaningful insights through visual exploration.

Towards this objective, we developed an automated pipeline to identify putative cell type–specific gene targets for each phenotype by integrating phenotype–cell type association results with primary resources such as GenCC gene–disease relationships and scRNA-seq atlas datasets, producing a table where each row represented a disease–phenotype–cell type–gene tetrad. We applied sequential filters to retain only significant phenotype–cell type associations ($FDR<0.05$), phenotype–gene pairs with strong causal evidence (GenCC score > 3), phenotypes with high specificity ($IC>8$), and gene–cell type links in the top 25% expression specificity quantile, and further required a symptom intersection > 0.25 when linking cell types to diseases via phenotypes. The filtered results were ranked by GPT-4 composite severity scores, with only the top 10 tetrads retained per phenotype, yielding compact, high-confidence networks suitable for manual inspection and visualization.

This yielded putative therapeutic targets for 5,252 phenotypes across 4,819 diseases in 201 cell types and 3,148 genes (Additional file 1: Fig. S6). While this constitutes a large number of genes in total, each phenotype was assigned a median of 2 gene targets (mean=$3.3$, min=1, max=10). Relative to the number of genes annotations per phenotype in the HPO overall (median=$7.0$, mean=$62$, min=1, max=5,003) this represents a substantial decrease in the number of candidate target genes, even when excluding high-level phenotypes (HPO level>3). It is also important to note that the phenotypes in the prioritised targets list are ranked by their severity, allowing us to distinguish between phenotypes with a high medical urgency (e.g. ‘Hydranencephaly’) from those with lower medical urgency (e.g. ‘Fair hair’). This can be useful for clinicians, biomedical scientists, and pharmaceutical manufacturers who wish to focus their research efforts on phenotypes with the greatest need for intervention.

Across all phenotypes, epithelial cells were most commonly implicated (838 phenotypes), followed by stromal cells (626 phenotypes), neurons (475 phenotypes), chondrocytes (383 phenotypes), endothelial cells (361 phenotypes), and fibroblasts (348 phenotypes). Grouped by higher-order ontology category, ‘Abnormality of the musculoskeletal system’ had the greatest number of enriched phenotypes (959 phenotypes, 857 genes), followed by ‘Abnormality of the nervous system’ (733 phenotypes, 1,138 genes), ‘Abnormality of head or neck’ (543 phenotypes, 986 genes), ‘Abnormality of the genitourinary system’ (443 phenotypes, 695 genes), and ‘Abnormality of the eye’ (377 phenotypes, 545 genes).

### Therapeutic target validation

To determine whether the genes prioritised by our therapeutic targets pipeline were plausible, we checked what percentage of existing gene therapy targets we recapitulated. Data on therapeutic approval status was gathered from the Therapeutic Target Database (TTD; release 2026-06−02) [23]. Overall, we prioritised $87$% (120 total) of all non-failed existing gene therapy targets (i.e. those which are currently approved, investigative, or undergoing clinical trials). A hypergeometric test confirmed that our prioritised targets were significantly enriched for non-failed gene therapy targets ($p=$ $5.6 \times 10^{-5}$, $\text {odds ratio=}$ $3.0$, $\text {sensitivity}$=$0.83$, $\text {specificity}$=$0.38$, Additional file 2: Table S11). For these hypergeometric tests, the background gene set was composed of the union of all phenotype-associated genes in the HPO and all gene therapy targets listed in TTD.

Even when considering therapeutics of any kind (Additional file 1: Fig. S7), not just gene therapies, we recapitulated $40$% of the non-failed therapeutic targets and 0% of the terminated/withdrawn therapeutic targets ($n=1,255$). Here we found that our prioritised targets were significantly depleted for failed therapeutics ($p=$ $4.4 \times 10^{-23}$, $\text {odds ratio=}$ $0.36$, $\text {sensitivity}$=$0.27$, $\text {specificity}$=$0.49$). This suggests that our multi-scale evidence-based prioritisation pipeline is capable of selectively identifying genes that are likely to be effective therapeutic targets.

In addition to aggregate enrichment results, we also provide specific examples of successful gene therapies whose cell type-specific mechanism were recapitulated by our phenotype-cell associations. In particular, our pipeline nominated the gene *RPE65* within ‘retinal pigment epithelial cells’ as the top target for ‘Fundus atrophy’ vision-related phenotypes that are hallmarks of ‘Leber congenital amaurosis, type II’ and ‘Severe early-childhood-onset retinal dystrophy’. Indeed, gene therapies targeting *RPE65* within the retina of patients with these rare genetic conditions are some of the most successful clinical applications of this technology to date, able to restore vision in many cases [52]. In other cases, a tissue (e.g. liver) may be known to be causally involved in disease genesis, but the precise causal cell types within that tissue remain unknown (e.g. hepatocytes, Kupffer cells, Cholangiocytes, Hepatic stellate cells, Natural killer cells, etc.). Tissue-level investigations (e.g. using bulk transcriptomics or epigenomics) would be dominated by hepatocytes, which comprise 75% of the liver. Our prioritized gene therapy targets can aid in such scenarios by providing the cell type-resolution context most likely to be causal for a given phenotype or set of phenotypes (Fig. 8).

### Selected example targets

From our prioritised targets, we selected four phenotype or disease examples: ‘GM2-ganglioside accumulation’, ‘Spinocerebellar atrophy’, ‘Neuronal loss in central nervous system’. To focus on clinically relevant phenotypes and reduce overplotting, we limited selection to those with GPT severity scores above $15$ (Additional file 1: Fig. S9). Selection was based on severity and network simplicity to allow compact visualisation.

Tay-Sachs disease (TSD) is a fatal neurodegenerative condition caused by *HEXA* deficiency and ganglioside buildup. We identified alternatively activated macrophages as the cell type most associated with ‘GM2-ganglioside accumulation’ (Additional file 1: Fig. S9). This aligns with prior findings of ganglioside accumulation in TSD macrophages [53–56]. Our results support macrophages as causal in TSD and the most promising therapeutic target.

Spinocerebellar atrophy is a progressive neurodegenerative phenotype in disorders like Spinocerebellar ataxia. Our pipeline implicates M2 macrophages (‘Alternatively activated macrophages’) as the only causal cell type (Additional file 1: Fig. S9). This suggests Purkinje cell loss is downstream of macrophage dysfunction, consistent with microglial roles in neurodegeneration [57–59]. Our findings provide the first statistically supported link between risk genes and this cell type, which is supported by relevant mouse models (e.g. *Atxn1*, *Pnpla6*) that replicate cellular and behavioural disease phenotypes.

‘Neuronal loss in central nervous system’ is a phenotype caused by multiple serious diseases (e.g. Huntington disease, frontotemporal lobar degeneration, and certain mitochondrial disorders). Across all of these diverse conditions with varying genetic causes (>8 genes), they converge on just 2 cell types: M2 macrophages and epithelial cells.

Additional examples of therapeutic targets include: cardiac muscle and endothelial cells in phenotypes associated with respiratory failure (Additional file 1: Fig. S10), microglia in frontal lobe dementia (Additional file 1: Fig. S11), chondrocytes in lethal skeletal dysplasia (Additional file 1: Fig. S12), endothelial cells in small vessel disease (Additional file 1: Fig. S13), oligodendrocytes and neurons in Parkinson’s disease (Additional file 1: Fig. S14), and multiple gastrointestinal and immune cell types in Alzheimer’s disease (Additional file 1: Fig. S15). For a more extensive discussion of these phenotypes, please refer to the Supplementary Results.

### Mappings

Mappings from HPO phenotypes and other commonly used medical ontologies (SNOMED, UMLS, ICD-9, and ICD-10) were gathered using the Ontology Xref Service (OxO; https://www.ebi.ac.uk/spot/oxo/) to facilitate others using our results in future work. Direct mappings, with a cross-ontology distance of 1, are the most precise and reliable. Counts of mappings at each distance are shown in Additional file 2: Table S1. In total, there were 15,105 direct mappings between the HPO and other ontologies, with the largest number of mappings coming from the UMLS ontology (12,898 UMLS terms).

## Discussion

Investigating rare diseases (RDs) at the phenotype level offers advantages in research and clinical medicine. Most RDs have a single known causal gene (7,671/8,631 = 89%), prohibiting traditional gene set enrichment-based analyses. Instead, aggregating genes into phenotype-based sets enables well-powered analyses (mean $\sim$ ${76}$ genes/phenotype). Phenotypes often converge on shared molecular pathways, and a phenotype-centric approach captures interindividual variation in disease presentations. This requires mapping the molecular and cellular mechanisms behind each phenotype, which we achieve here at phenome scale.

Across 201 cell types and 11,047 phenotypes, we found >46,514 significant phenotype–cell type relationships, enabling multi-scale mechanistic tracing of disease biology. Results replicate known links, add cellular context, and uncover novel associations. Extensive benchmarking confirmed expected associations, aided by comprehensive phenotype and cell type ontologies. Key findings include enrichment of anatomically matched associations, correlation of phenotype specificity with association strength, precise subtypes for recurrent infections, and links between congenital onset frequency and developmental cell types.

It is a matter of ongoing scientific debate as to whether the rare, more monogenic versions of diseases and phenotypes share the same genetic etiology as their common, polygenic counterparts [60, 61]. Nevertheless, we identified several examples where these two disparate data sources converge on the same cell types. These include the implication of smooth muscle cells and endothelial cells in small vessel disease [62–64], and the consistent association between macrophages and neurodegenerative diseases such as Alzheimer’s and Parkinson’s [62, 65–68]. Additionally, we recover genome-wide association study (GWAS) -derived associations between macular degeneration and specific cell types of the eye (photoreceptor cells, retinal pigment cells, Mueller cells), immune system (dendritic cells, macrophages), and vascular system (endothelial cells) [69–72].

Despite our growing knowledge of RD genetics, less than 5% of RDs have available treatments [6]. However, advances in CRISPR, prime editing, antisense oligonucleotides, and viral/lipid delivery [73–76] are accelerating. The FDA’s new program [77] aims to expand gene/cell therapy approvals in years rather than decades [78], but success depends on understanding the causal mechanisms of each RD. Here, we built a reproducible pipeline for nominating cell type–resolved therapeutic targets (Fig. 9), factoring in association strength, gene specificity, severity, therapy delivery suitability, and model translatability. We recovered $87$% of active gene therapies, confirming strong enrichment. Highlighted cases include macrophage-driven phenotypes in Tay-Sachs, spinocerebellar ataxia, and Alzheimer’s disease, pinpointing links between specific phenotypes (e.g. neurofibrillary tangles) and causal cell types.

Current limitations of our study include missing certain rare cell subtypes and states (e.g. immune cell responses, diseased states, aging) and incomplete knowledge of gene–phenotype associations. A related concern is that our statistical power to detect cell type associations may be diminished in phenotypes with very few gene associations. Fewer genes decreases variance explained with respect to the denser cell type specificity vectors, in turn decreasing power. The remaining associations for the most specific phenotypes therefore reflect cases where the effect size is sufficiently strong to overcome the reduced variance (Fig. 3). Weaker cell type associations with highly monogenic phenotypes may be missed by our analyses. With the expectation that data will continue to improve over time, our pipeline is fully containerised and documented for end-to-end reproducibility. Comprehensive, ontology-aligned frameworks such as ours enable discovery, diagnosis, and basket trial design for shared molecular etiologies across many diseases [79]. Furthermore, we invite collaborations to validate and translate these predictions, and have publicly released all results via R packages and the Rare Disease Celltyping Portal (https://neurogenomics-ukdri.dsi.ic.ac.uk/) to support broad access for researchers, clinicians, and patients.

## Conclusions

We present a scalable, cost-effective, and reproducible framework for phenome-wide, cell type-specific mechanism prediction in rare diseases. By integrating the Human Phenotype Ontology with whole-body single-cell transcriptomic atlases spanning embryonic, foetal, and adult stages, we systematically nominate causal cell types for thousands of phenotypes and prioritise candidate gene-therapy targets across the rare-disease landscape. Coupled with continuing advances in gene therapy and supportive regulatory changes, this approach can help realise the promise of genomic medicine for the global rare-disease community.

## Supplementary information

Additional file 1. Supplementary figuresand supplementary tables, each with its full legend

Additional file 2. Supplementary tablesin machine-readable form

## Data Availability

- Human Phenotype Ontology: https://hpo.jax.org - GenCC: https://thegencc.org - Descartes Human scRNA-seq atlas: https://cellxgene.cziscience.com/collections/c114c20f-1ef4-49a5-9c2e-d965787fb90c - Human Cell Landscape scRNA-seq atlas: https://cellxgene.cziscience.com/collections/38833785-fac5-48fd-944a-0f62a4c23ed1 - Processed Cell Type Datasets (ctd_DescartesHuman.rds and ctd_HumanCellLandscape.rds): https://github.com/neurogenomics/MSTExplorer/releases - Gene x Phenotype association matrix (hpo_matrix.rds): https://github.com/neurogenomics/MSTExplorer/releases - GPT-4 phenotype severity annotations: https://github.com/neurogenomics/rare_disease_celltyping/releases/download/latest/gpt_check_annot.csv.gz - Full phenotype-cell type association test results: https://github.com/neurogenomics/MSTExplorer/releases/download/v0.1.10/phenomix_results.tsv.gz - Rare Disease Celltyping Portal: https://neurogenomics-ukdri.dsi.ic.ac.uk - Rare Disease Celltyping Portal data: https://zenodo.org/records/15147825.
