# Supplementary material for: Cell type-specific contextualisation of the human phenome: towards the systematic treatment of all rare diseases
Source: Genome Med. 2026 Jun 26;18:113. doi: 10.1186/s13073-026-01692-0 (PMC13411100; doi:10.1186/s13073-026-01692-0)
Supplement: Supplementary file 1 — Additional file 1. Supplementary figuresand supplementary tables, each with its full legend [file 13073_2026_1692_MOESM1_ESM.pdf]

Supplementary Materials

Supplementary Results

Congenital phenotypes are associated with foetal cell types

We sought to test whether some cell types tend to show strong differences in their phenotype associations between their foetal and adult forms. To do this, we performed an analogous enrichment procedure as with the phenotypes, except using Cell Ontology terms and the Cell Ontology graph. This analysis identified the cell type category connective tissue cell ($p = 1.8 \times 10^{-3}$, log_2(fold-change)=3.2) as the most foetal-biased cell type. No cell type categories were significantly enriched for the most adult-biased cell types. This is likely due to the fact that cell types can be disrupted at different stages of life, resulting in different phenotypes. Thus the same cell types may be involved in both the most foetal-biased and adult-biased phenotypes.

Selected example targets

From our prioritised targets, we selected the following four sets of phenotypes or diseases as examples: 'GM2-ganglioside accumulation', 'Spinocerebellar atrophy', 'Neuronal loss in central nervous system'. Only phenotypes with a GPT severity score greater than 15 were considered to avoid overplotting and to focus on the more clinically relevant phenotypes Fig. 9a-h. These examples were then selected partly on the basis of severity rankings, and partly for their relatively smaller, simpler networks than lent themselves to compact visualisations.

Tay-Sachs disease (TSD) is a devastating hereditary condition in which children are born appearing healthy, which gradually degrades leading to death after 3-5 years. The underlying cause is the toxic accumulation of gangliosides in the nervous system due to a loss of the enzyme produced by *HEXA*. While this could in theory be corrected with gene editing technologies, there remain some outstanding challenges. One of which is identifying which cell types should be targeted to ensure the most effective treatments. Here we identified alternatively activated macrophages as the cell type most strongly associated with 'GM2-ganglioside accumulation' Fig. 9i. The role of aberrant macrophage activity in the regulation of ganglioside levels is supported by observation that gangliosides accumulate within macrophages in TSD[53], as well as experimental evidence in rodent models[54–56]. Our results not only corroborate these findings, but propose macrophages as the primary causal cell type in TSD, making it the most promising cell type to target in therapies.

Another challenge in TSD is early detection and diagnosis, before irreversible damage has occurred. Our pipeline implicated extravillous trophoblasts of the placenta in 'GM2-ganglioside accumulation'. While not necessarily a target for gene therapy (as the child is detached from the placenta after birth), checking these cells *in utero* for an absence of *HEXA* may serve as a viable biomarker as these cells normally express the gene at high levels. Early detection of TSD may lengthen the window of opportunity for therapeutic intervention[81], especially when genetic sequencing is not available or variants of unknown significance are

found within *HEXA*[82].

Spinocerebellar atrophy is a debilitating and lethal phenotype that occurs in diseases such as Spinocerebellar ataxia and Boucher-Neuhäuser syndrome. These diseases are characterised by progressive degeneration of the cerebellum and spinal cord, leading to severe motor and cognitive impairments. Our pipeline identified M2 macrophages (labeled as the closest CL term 'Alternatively activated macrophages' in Fig. 9j) as the only causal cell type associated with 'Spinocerebellar atrophy'. This strongly suggests that degeneration of cerebellar Purkinje cells are in fact downstream consequences of macrophage dysfunction, rather than being the primary cause themselves. This is consistent with the known role of macrophages, especially microglia, in neuroinflammation and other neurodegenerative conditions such as Alzheimer's and Parkinson's disease[57–59]. While experimental and postmortem observational studies have implicated microglia in spinocerebellar atrophy previously[57], our results provide a statistically-supported and unbiased genetic link between known risk genes and this cell type. Therefore, targeting M2 macrophages in the treatment of spinocerebellar atrophy may represent a promising therapeutic strategy. This is aided by the fact that there are mouse models that perturb the ortholog of human spinocerebellar atrophy risk genes (e.g. *Atxn1*, *Pnpla6*) and reliably recapitulate the effects of this disease at the cellular (e.g. loss of Purkinje cells), morphological (e.g. atrophy of the cerebellum, spinal cord, and muscles), and functional (e.g. ataxia) levels.

Next, we investigated the phenotype 'Neuronal loss in central nervous system'. Despite the fact that this is a fairly broad phenotype, we found that it was only significantly associated with 3 cell types (alternatively activated macrophage, macrophage, epithelial cell), specifically M2 macrophages and sinusoidal endothelial cells Fig. 9k.

Skeletal dysplasia is a heterogeneous group of over 450 disorders that affect the growth and development of bone and cartilage. This phenotype can be lethal when deficient bone growth leads to the constriction of vital organs such as the lungs. Even after surgical interventions, these complications continue to arise as the child develops. Pharmacological interventions to treat this condition have largely been ineffective. While there are various cell types involved in skeletal system development, our pipeline nominated chondrocytes as the causal cell type underlying the lethal form of this condition (Fig. S12). Assuringly, we found that the disease 'Achondrogenesis Type 1B' is caused by the genes *SLC26A2* and *COL2A1* via chondrocytes. We also found that 'Platyspondylic lethal skeletal dysplasia, Torrance type' was associated with chondrocytes via *SLC26A2* and *COL2A1*. Thus, in cases where surgical intervention is insufficient, targeting these genes within chondrocytes may prove a viable long-term solution for children suffering from lethal skeletal dysplasia.

Alzheimer's disease (AD) is the most common neurodegenerative condition. It is characterised by a set of variably penetrant phenotypes including memory loss, cognitive decline, and cerebral proteinopathy. Interestingly, we found that different forms of early onset AD (which are defined by the presence of a specific disease gene) are each associated with different cell types via different phenotypes (Fig. S15). For example, AD 3 and AD 4 are primarily associated with cells of the digestive system ('enterocyte', 'gastric goblet

cell') and are implied to be responsible for the phenotypes 'Senile plaques', 'Alzheimer disease', 'Parietal hypometabolism in FDG PET'. Meanwhile, AD 2 is primarily associated with immune cells ('alternatively activated macrophage') and is implied to be responsible for the phenotypes 'Neurofibrillary tangles', 'Long-tract signs'. This suggests that different forms of AD may be driven by different cell types and phenotypes, which may help to explain its variability in onset and clinical presentation.

Finally, Parkinson's disease (PD) is characterised by motor symptoms such as tremor, rigidity, and bradykinesia. However there are a number of additional phenotypes associated with the disease that span multiple physiological systems. PD 19a and PD 8 seemed to align most closely with the canonical understanding of PD as a disease of the central nervous system in that they implicated oligodendrocytes and neurons (Fig. S14). Though the reference datasets being used in this study were not annotated at sufficient resolution to distinguish between different subtypes of neurons, in particular dopaminergic neurons. PD 19a/8 also suggested that risk variants in *LRRK2* mediate their effects on PD through both myeloid cells and oligodendrocytes by causing gliosis of the substantia nigra. The remaining clusters of PD mechanisms revolved around chondrocytes (PD 20), amacrine cells of the eye (hereditary late-onset PD), and the respiratory/immune system (PD 14). While the diversity in cell type-specific mechanisms is somewhat surprising, it may help to explain the wide variety of cross-system phenotypes frequently observed in PD.

It should be noted that the HPO only includes gene annotations for the monogenic forms of AD and PD. However it has previously been shown that there is at least partial overlap in their phenotypic and genetic etiology with respect to their common forms. Thus understanding the monogenic forms of these diseases may shed light onto their more common counterparts.

Experimental model translatability

We computed interspecies translatability scores using a combination of both ontological (SIM_o) and genotypic (SIM_g) similarity relative to each homologous human phenotype and its associated genes Fig. S8. In total, we mapped 281 non-human phenotypes (in *Caenorhabditis elegans*, *Danio rerio*, *Mus musculus*, *Rattus norvegicus*) to 375 homologous human phenotypes. Amongst the 5,252 phenotype within our prioritised therapy targets, 154 had viable animal models in at least one non-human species. Per species, the number of homologous phenotypes was: *Mus musculus* (n=146), *Danio rerio* (n=29), *Rattus norvegicus* (n=5), *Caenorhabditis elegans* (n=1). Amongst our prioritised targets with a GPT-4 severity score of >10, the phenotypes with the greatest animal model similarity were 'Odontodysplasia' ($SIM_{og} = 0.97$), 'Wide-cupped costochondral junctions' ($SIM_{og} = 0.93$), 'Short tubular bones of the hand' ($SIM_{og} = 0.85$), 'Megacystis' ($SIM_{og} = 0.76$), 'Cryptophthalmos' ($SIM_{og} = 0.71$).

Supplementary Figures

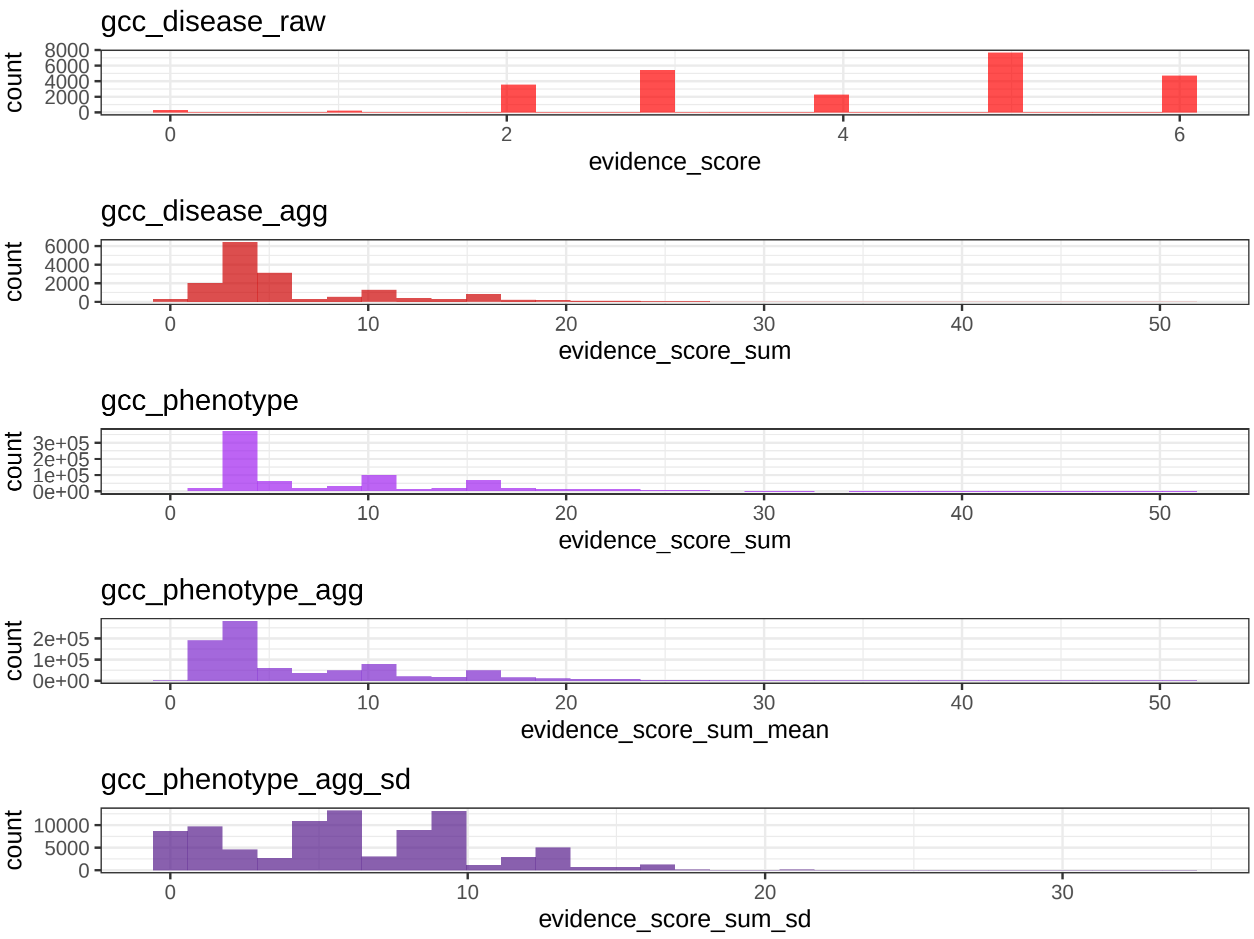

Figure S1: **Distribution of GenCC evidence scores at each processing step.** GenCC (https://thegencc.org/) is a database where semi-quantitative scores for the current strength of evidence attributing disruption of a gene as a causal factor in a given disease. "gcc_disease_raw" is the distribution of raw GenCC scores before any aggregation. "gcc_disease_agg" is the distribution of GenCC scores after aggregating by disease. "gcc_phenotype" is the distribution of scores after linking each phenotype to one or more disease. "gcc_phenotype_agg" is the distribution of scores after aggregating by phenotype, while "gcc_phenotype_agg_sd" is the standard deviation of those aggregated scores.

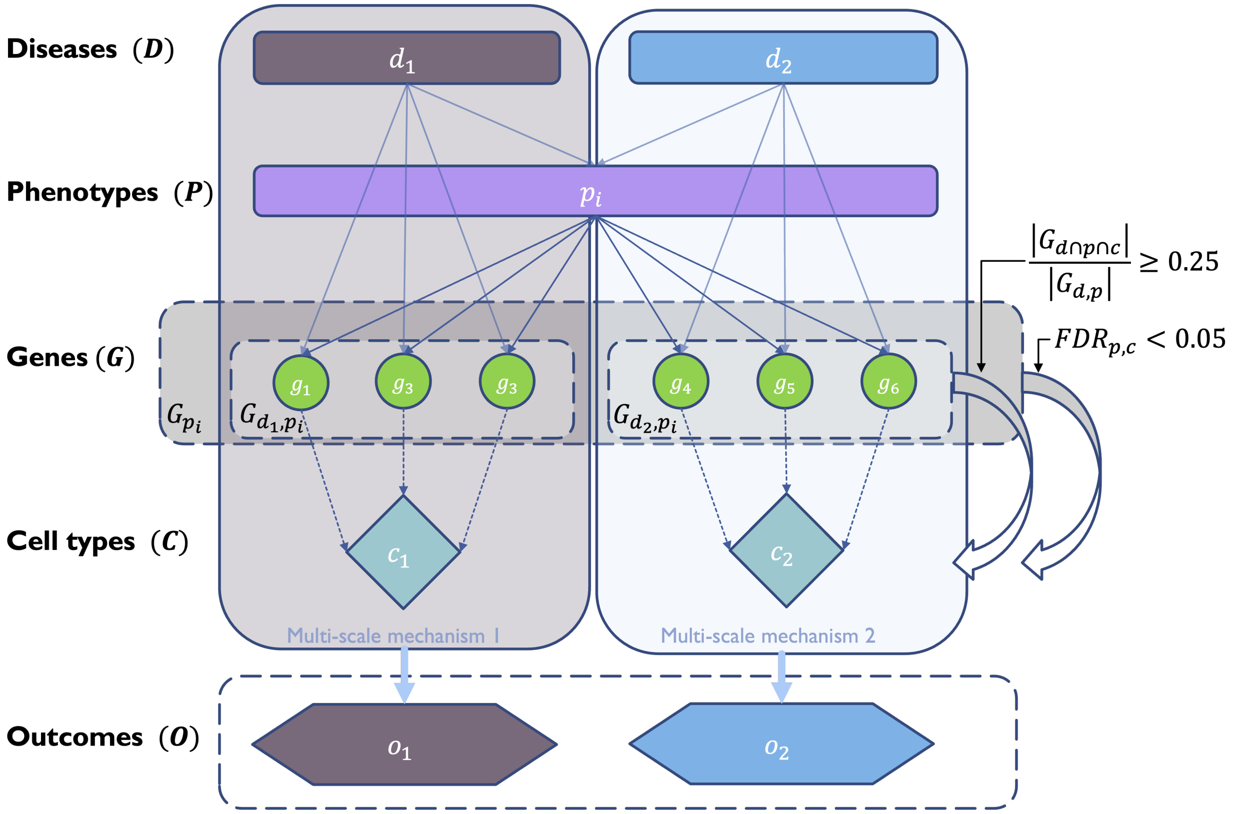

Figure S2: **Diagrammatic overview of multi-scale disease investigation strategy.** Here we provide an abstract example of differential disease etiology across multiple scales: diseases (D), phenotypes (P), cell types (C), genes (G), and clinical outcomes (O). In the HPO, genes are assigned to phenotypes via particular diseases (G_{dp}). Therefore, the final gene list for each phenotype is aggregated from across multiple diseases (G_p). We performed association tests for all pairwise combinations of cell types and phenotypes and filtered results after multiple testing corrections (FDR<0.05). Each phenotype in the context of a given disease is referred to here as a symptom. Links were established between symptoms and cell types through proportional gene set overlap at a minimum threshold of 25%.

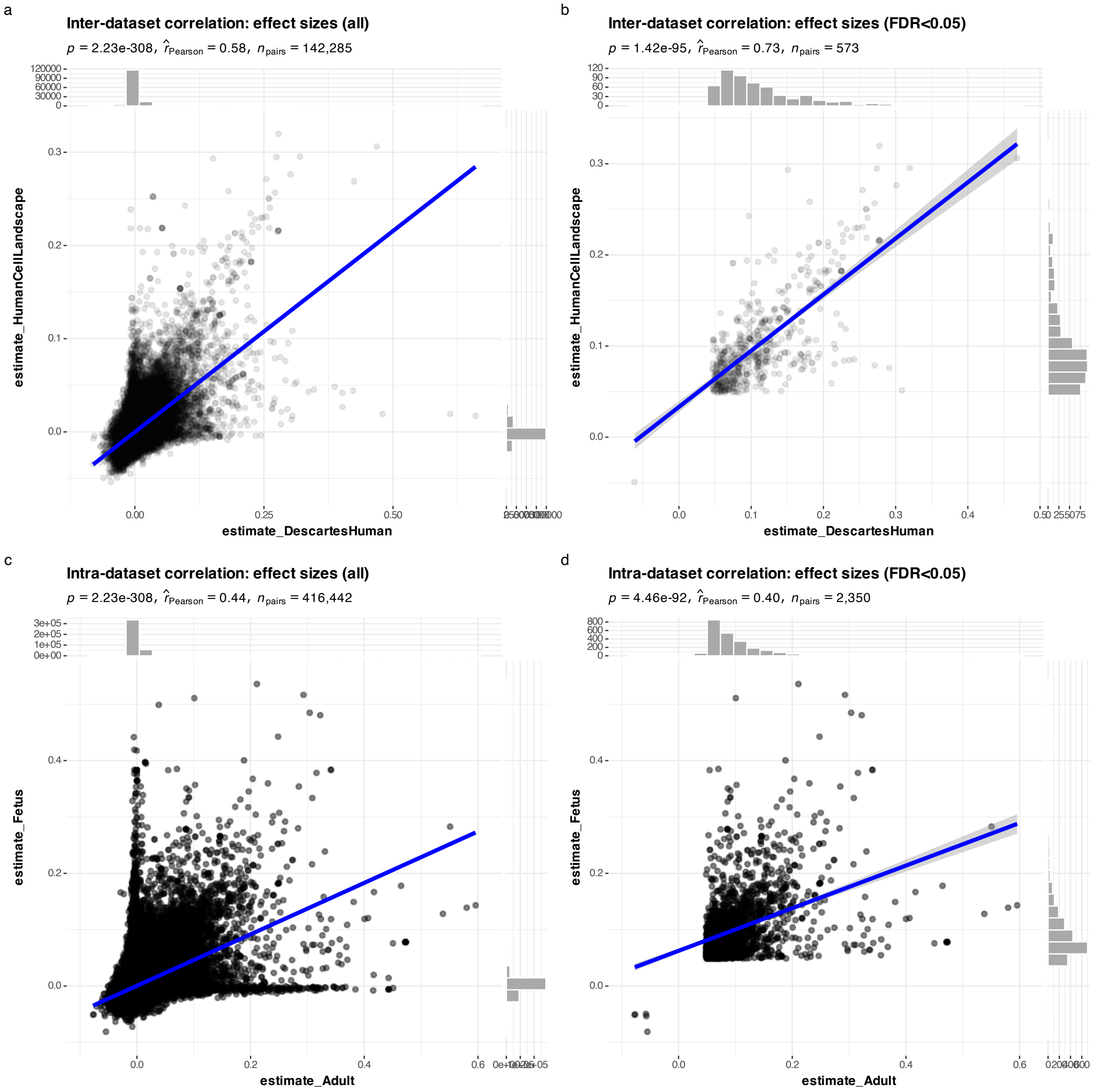

Figure S3: **Inter- and intra-dataset validation across the different CellTypeDataset (CTD) and developmental stages.** Correlations are computed using Pearson correlation coefficient. Point density is plotted using a 2D kernel density estimate. **a** Correlation between the uncorrected p-values from all phenotype-cell type association tests using the Descartes Human vs. Human Cell Landscape CTDs. **b** Correlation between the $log_{10}(fold - change)$ from significant phenotype-cell type association tests (FDR<0.05) using the Descartes Human vs. Human Cell Landscape CTDs. **c** Correlation between the uncorrected p-values from all phenotype-cell type association tests using the Human Cell Landscape foetal samples vs. Human Cell Landscape adult samples. **d** Correlation between the $log_{10}(fold - change)$ from significant phenotype-cell type association tests (FDR<0.05) using the Human Cell Landscape foetal samples vs. Human Cell Landscape adult samples.

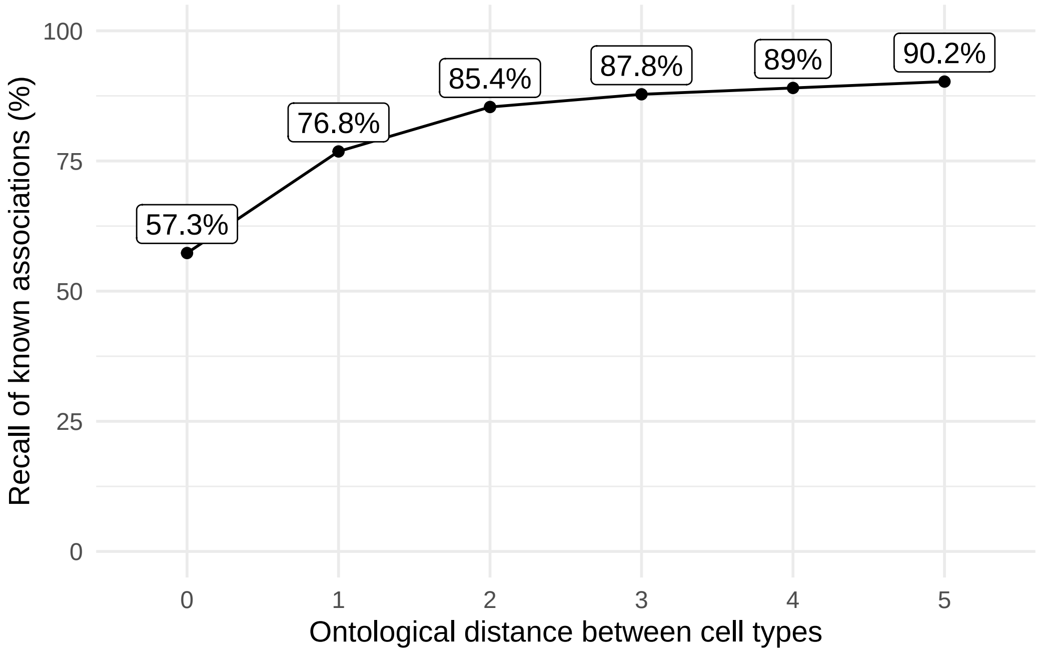

Figure S4: **Recall of ground-truth Monarch Knowledge Graph phenotype-cell type relationships at each ontological distance between cell types according to the Cell Ontology.**

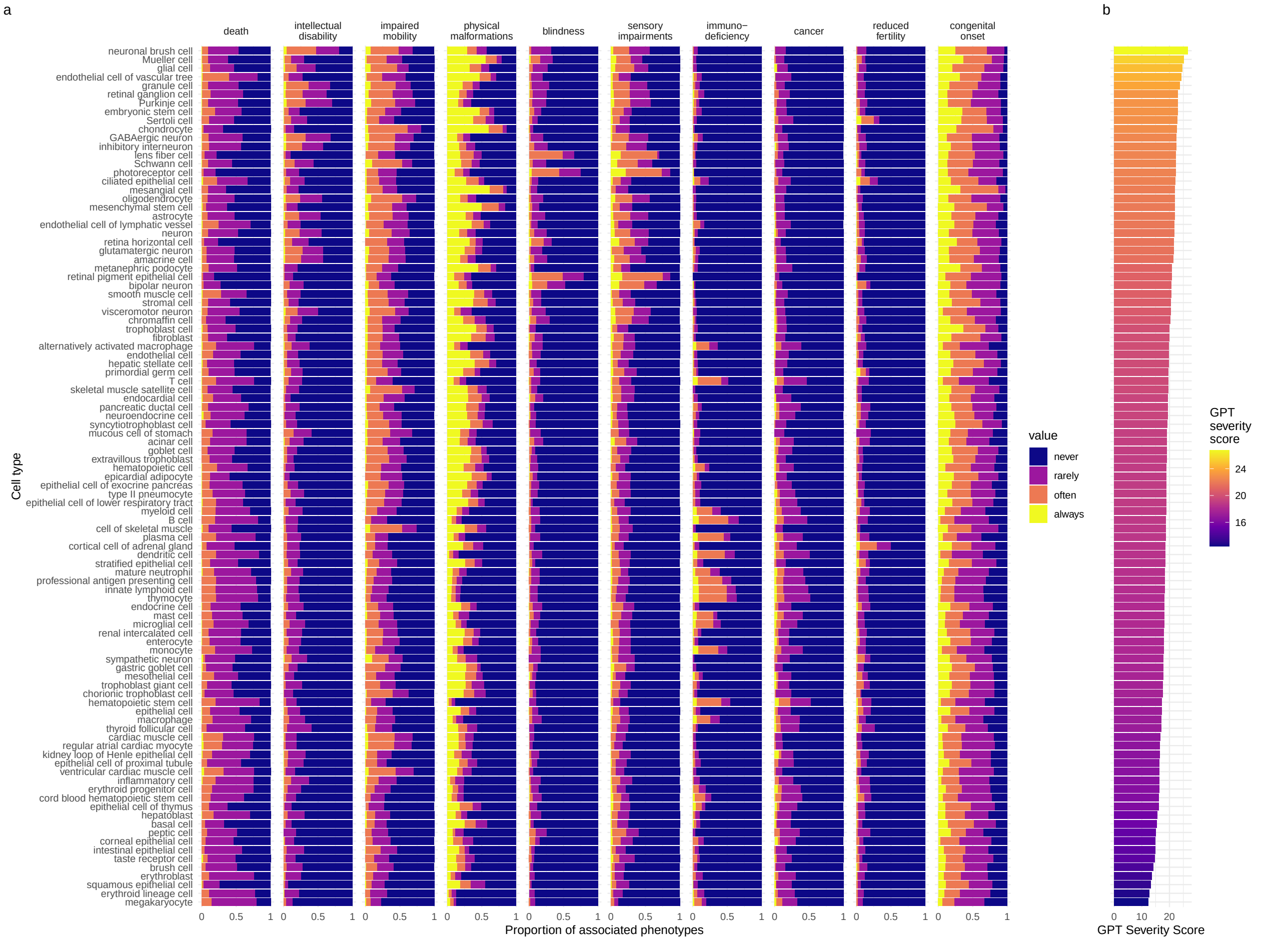

Figure S5: **Cell types ordered by the mean severity of the phenotypes they're associated with. a**, The distribution of phenotype severity annotation frequencies aggregated by cell type. **b**, The composite severity score, averaged across all phenotypes associated with each cell type.

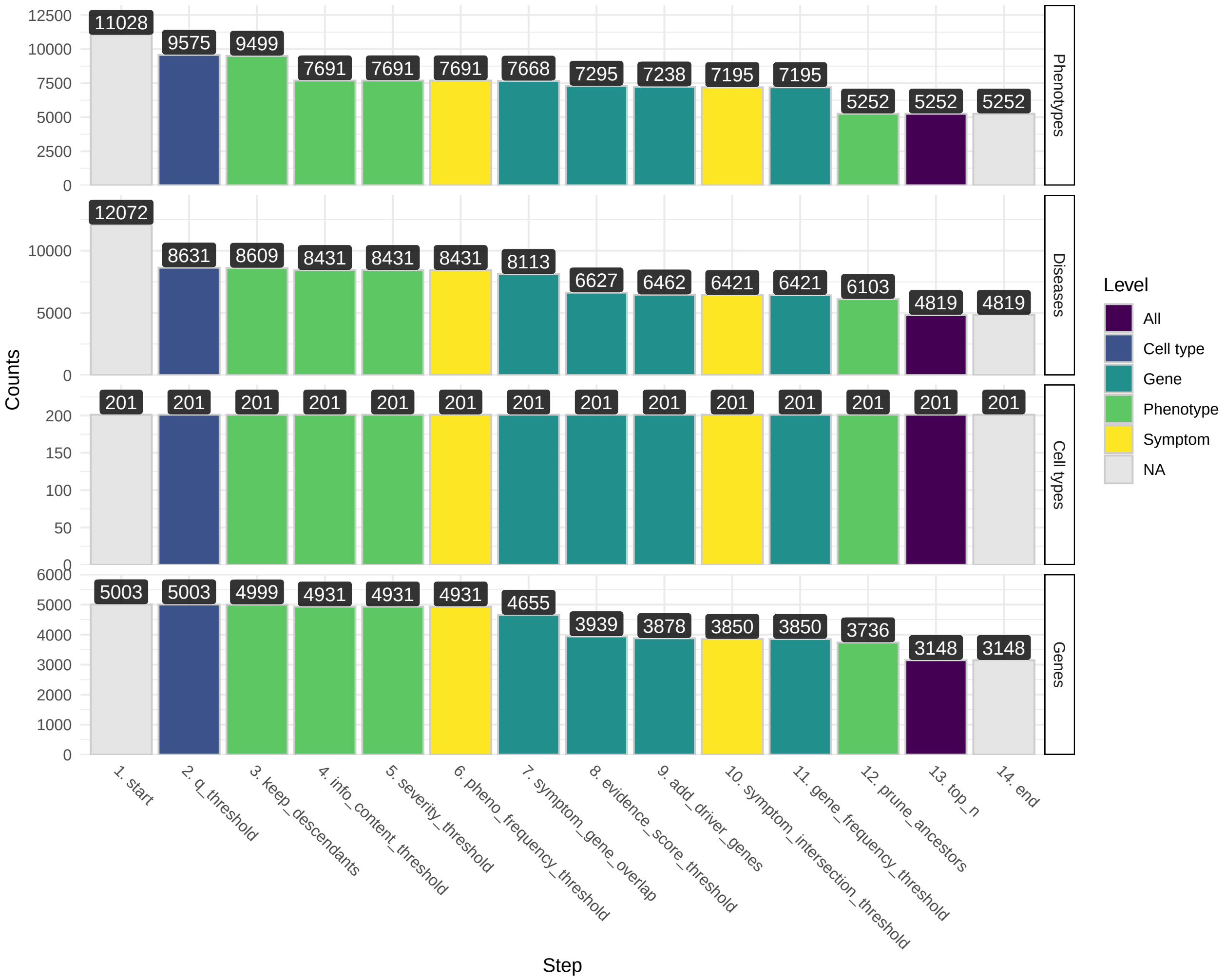

Figure S6: **Prioritised target filtering steps.** This plot visualises the number of unique phenotype-cell type associations, cell types, genes, and phenotypes (*y-axis*) at each filtering step (*x-axis*) within the multi-scale therapeutic target prioritisation pipeline. Each step in the pipeline can be easily adjusted according to user preference and use case. See Table S3 for descriptions and criterion of each filtering step.

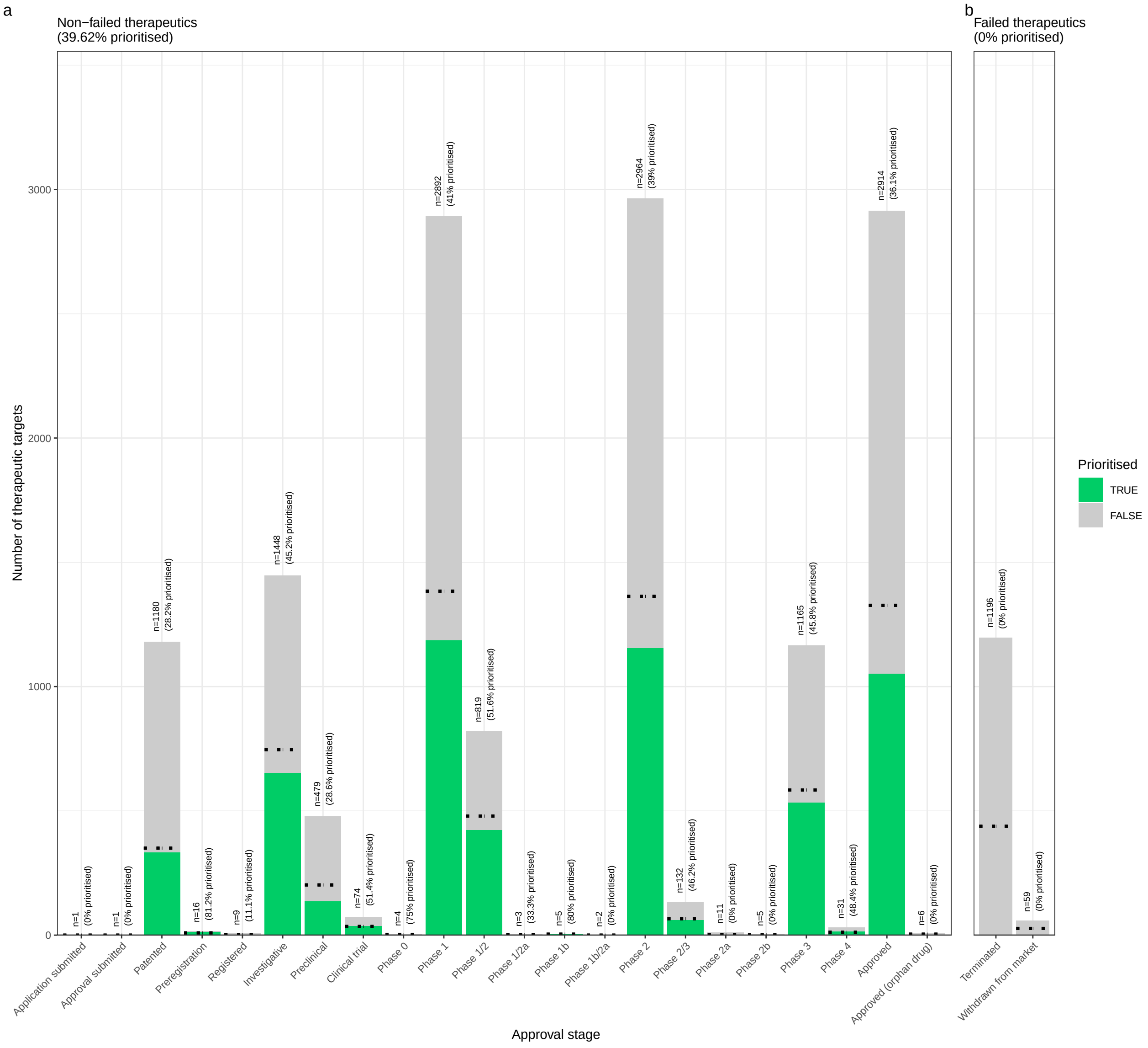

Figure S7: **Validation of prioritised therapeutic targets.** Proportion of existing all therapy targets (documented in the Therapeutic Target Database) recapitulated by our prioritisation pipeline.

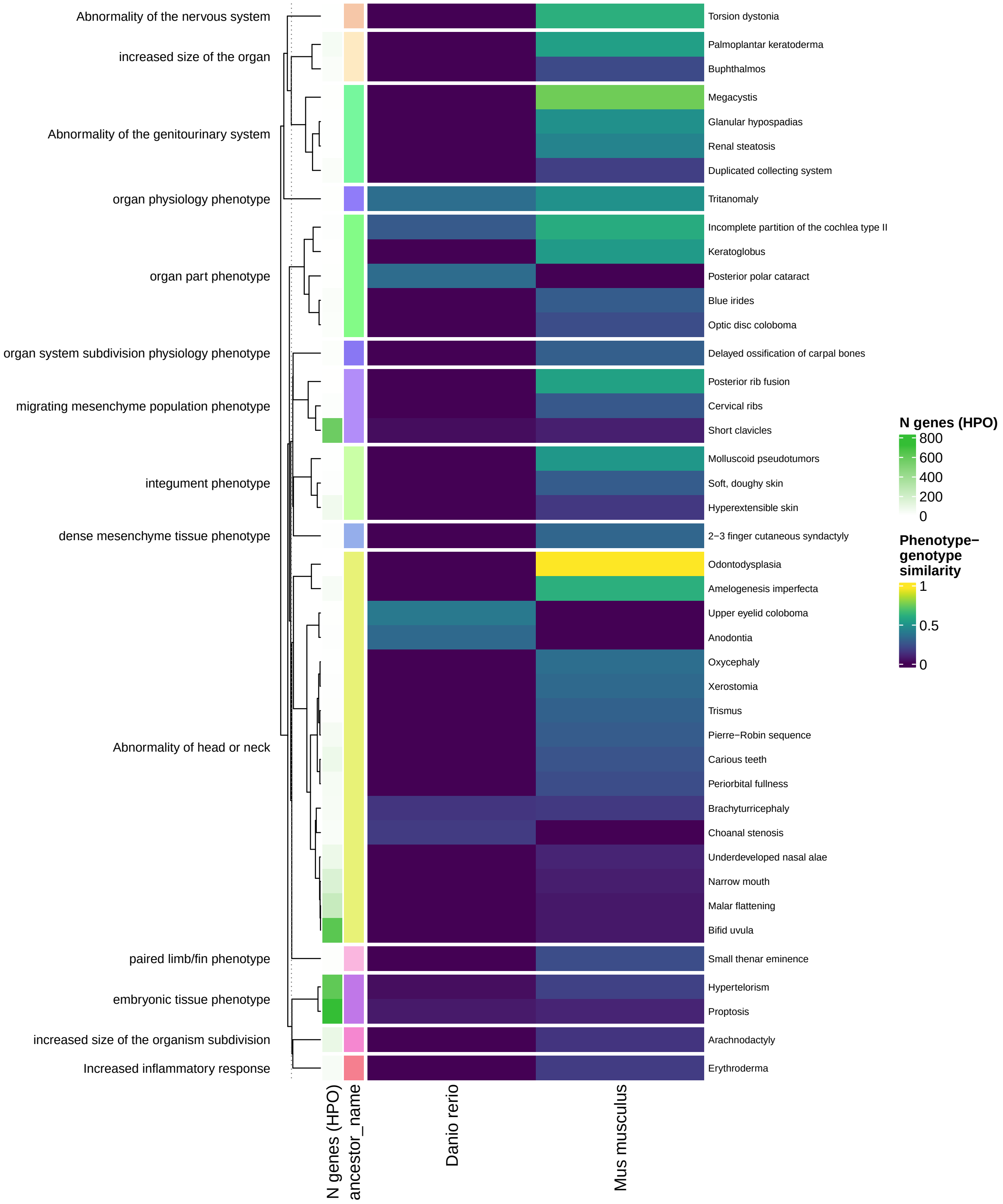

Figure S8: **Identification of translatable experimental models.** Interspecies translatability of the top human phenotypes nominated by the gene therapy prioritised pipeline. Above, the combined ontological-genotypic similarity score (SIM_{og}) is displayed as the heatmap fill colour stratified by the model organism (*x-axis*). An additional column ("n_genes_db1" on the far left) displays the total number of unique genes annotated to the phenotype within the HPO. Phenotypes are clustered according to their ontological similarity in the HPO (*y-axis*).

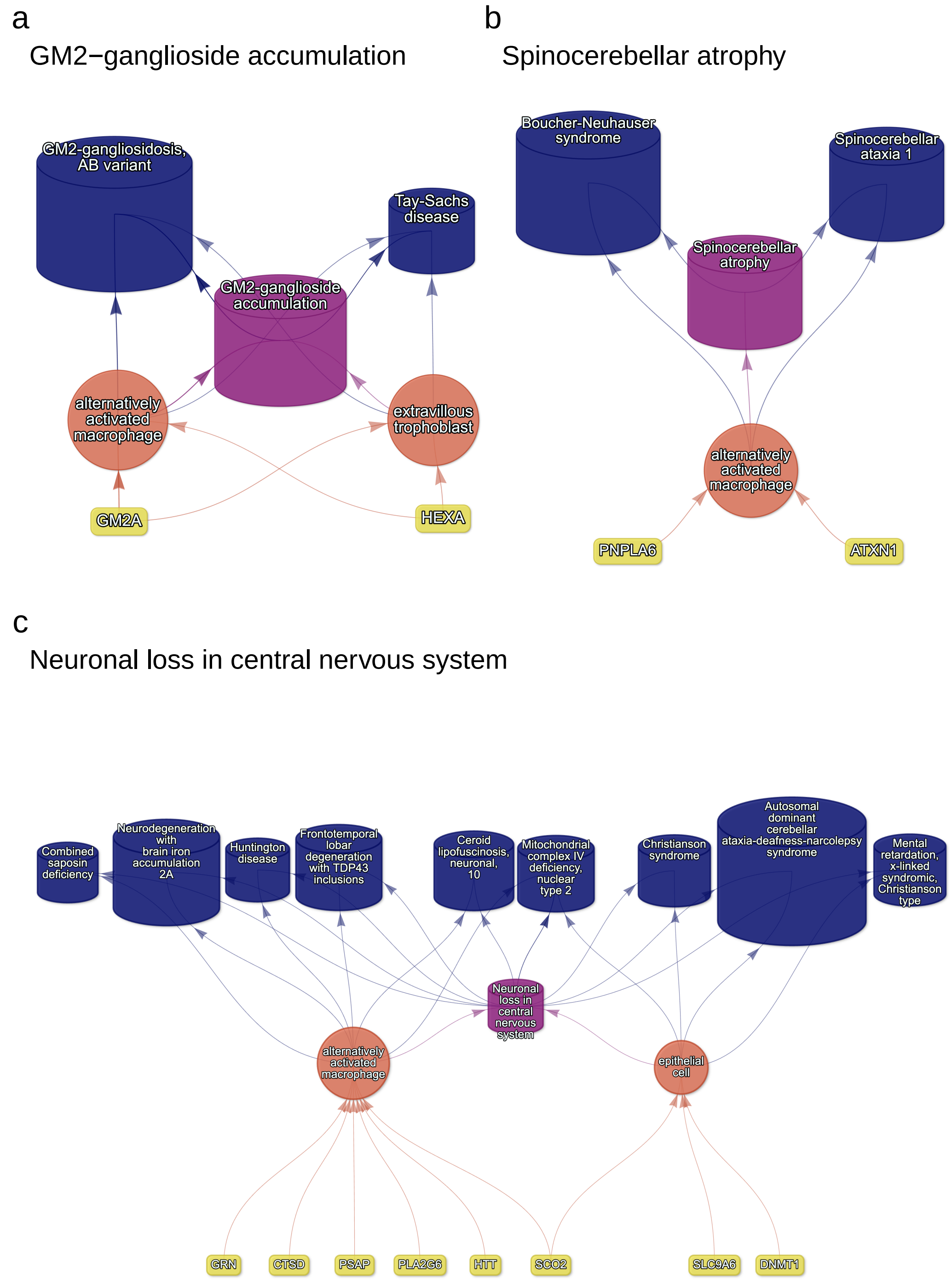

Figure S9: **Causal multi-scale networks reveal cell type-specific therapeutic targets.** Each disease (blue cylinders) is connected to its phenotype (purple cylinders) based on well-established clinical observations recorded within the HPO[11]. Phenotypes are connected to cell types (orange circles) via association testing between weighted gene sets (FDR<0.05). Each cell type is connected to the prioritised gene targets (yellow boxes) based on the driver gene analysis.The thickness of the edges connecting the nodes represent the (mean) fold-change from the bootstrapped enrichment tests. Nodes were spatially arranged using the Sugiyama algorithm[47].

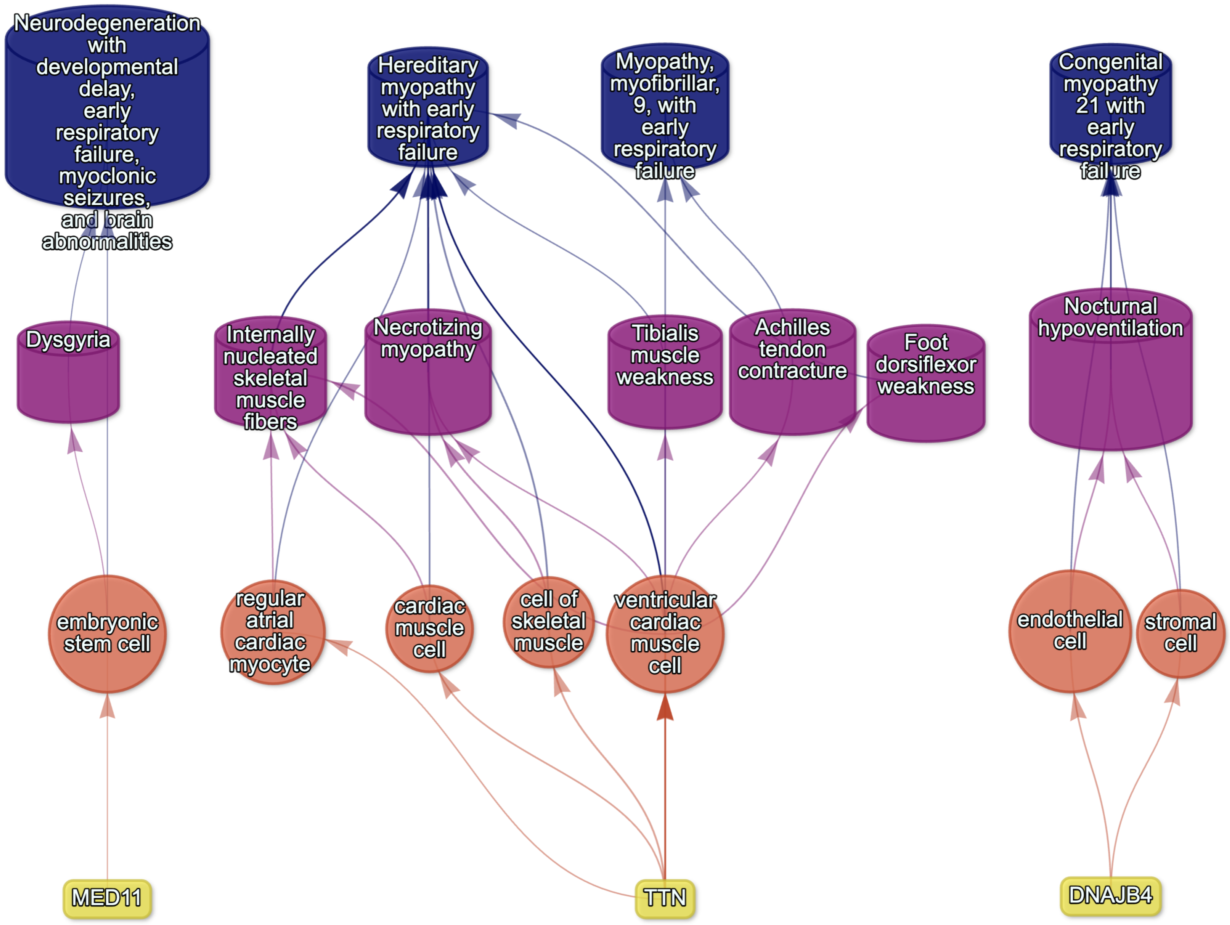

Figure S10: **Example cell type-specific gene therapy targets for phenotypes associated with respiratory failure-related diseases.** Each disease (*blue cylinders*) is connected to its phenotype (*purple cylinders*) based on well-established clinical observations recorded within the HPO[11]. Phenotypes are connected to cell types (*red circles*) via association testing between weighted gene sets (FDR<0.05). Each cell type is connected to the prioritised gene targets (*yellow boxes*) based on the driver gene analysis. The thickness of the edges connecting the nodes represent the (mean) fold-change from the bootstrapped enrichment tests. Nodes were spatially arranged using the Sugiyama algorithm[47].

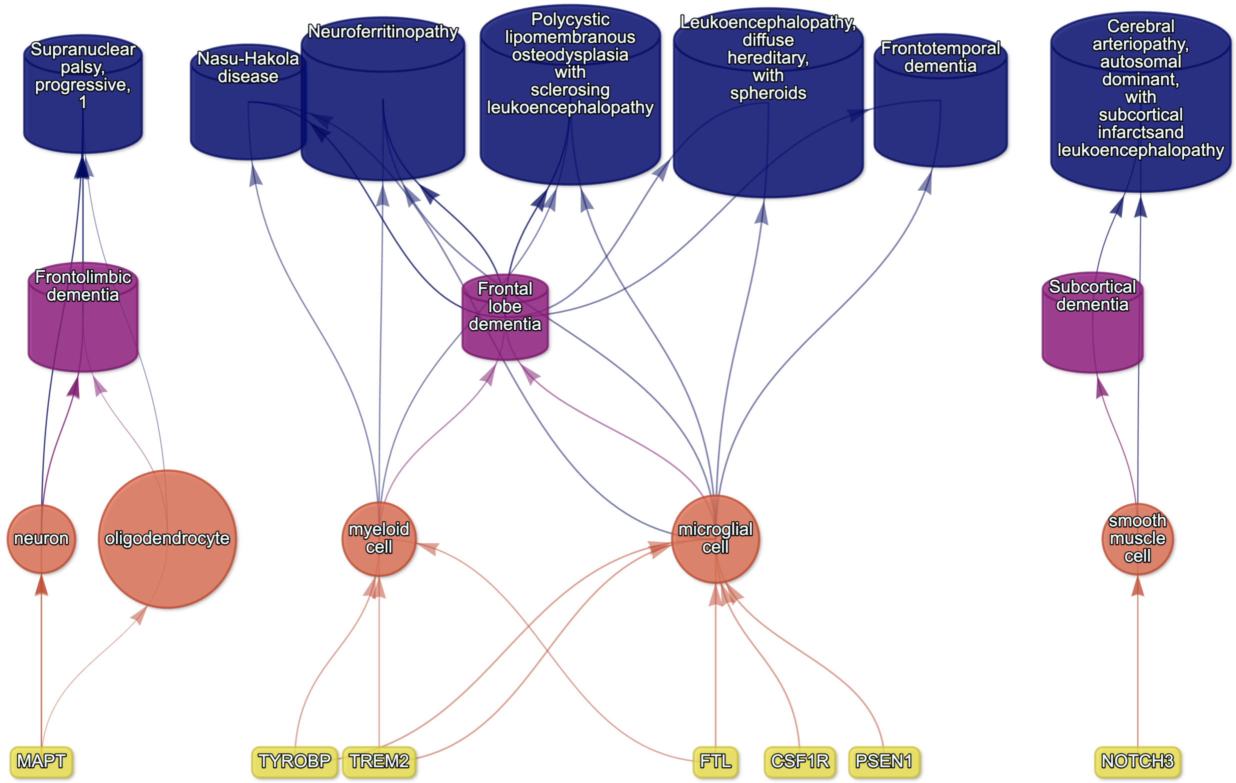

Figure S11: **Causal multi-scale network for dementia phenotypes.**

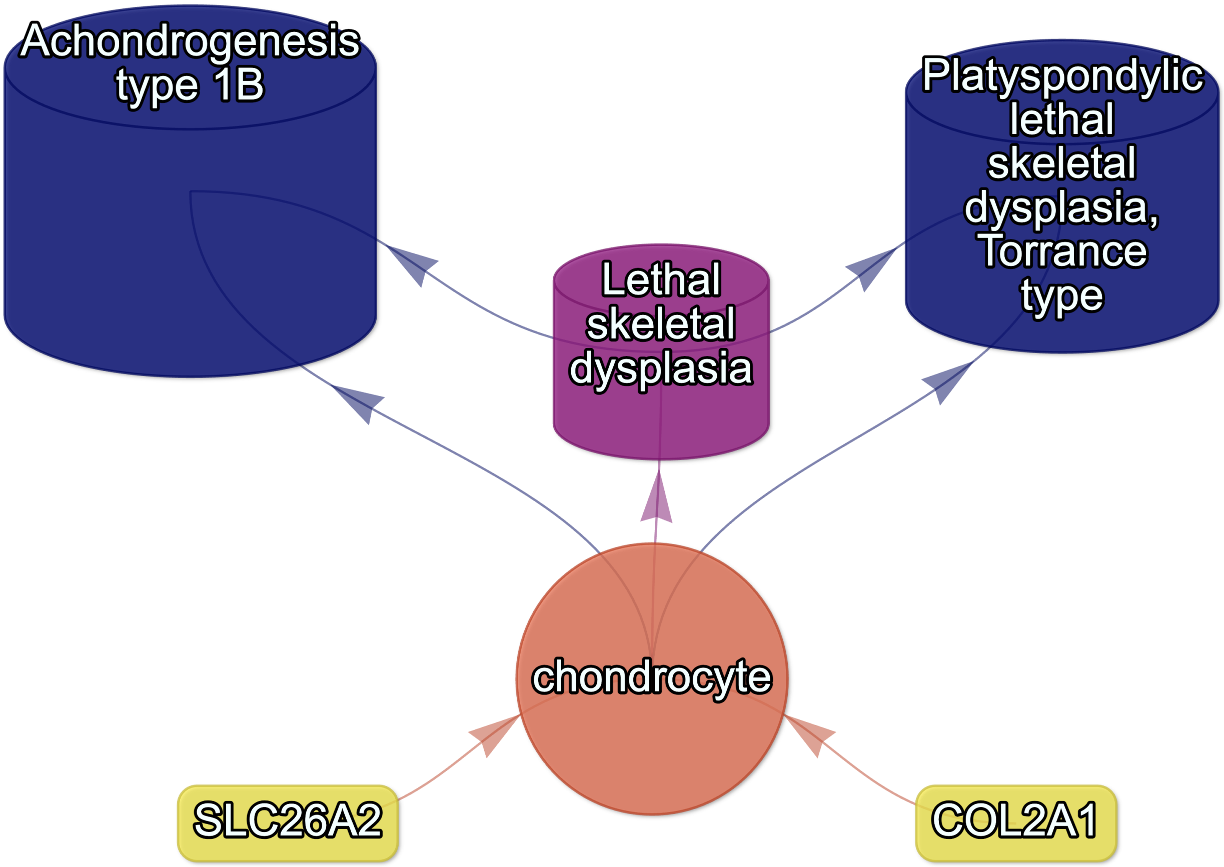

Figure S12: **Causal multi-scale network for the phenotype lethal skeletal dysplasia.**

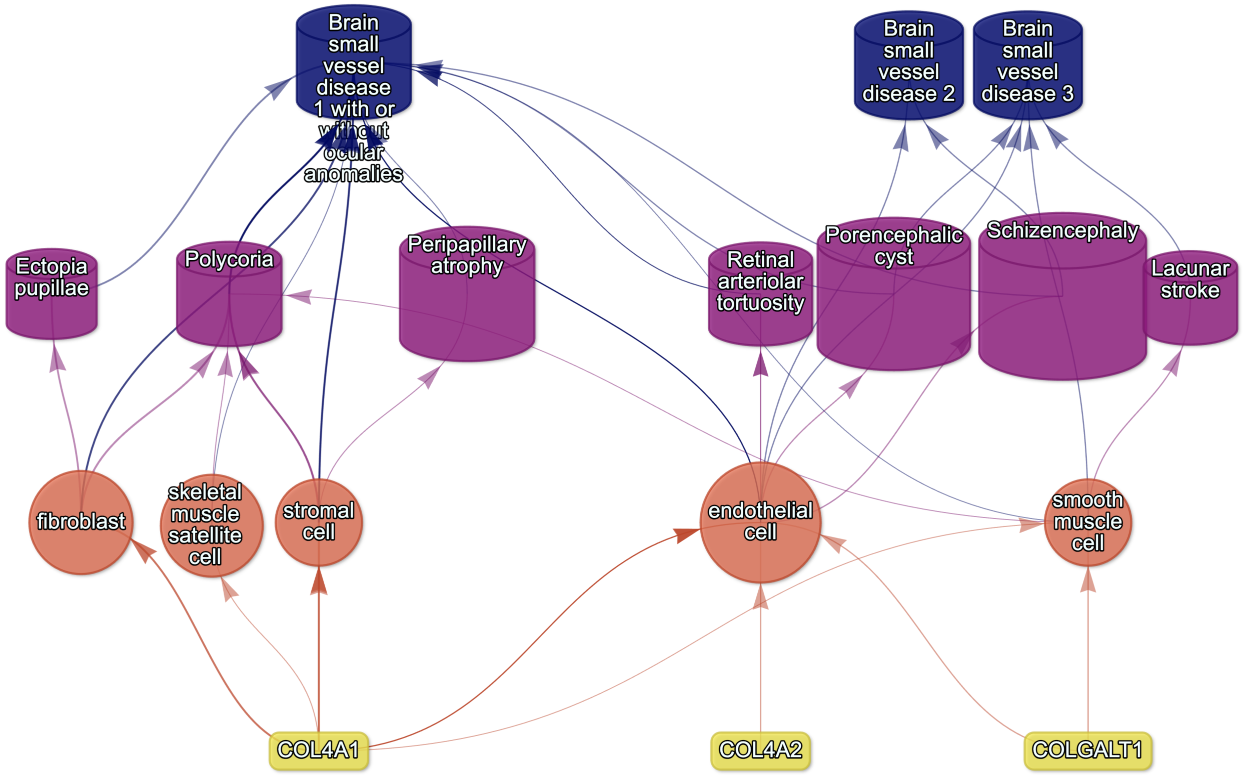

Figure S13: **Causal multi-scale network for phenotypes associated with small vessel disease.**

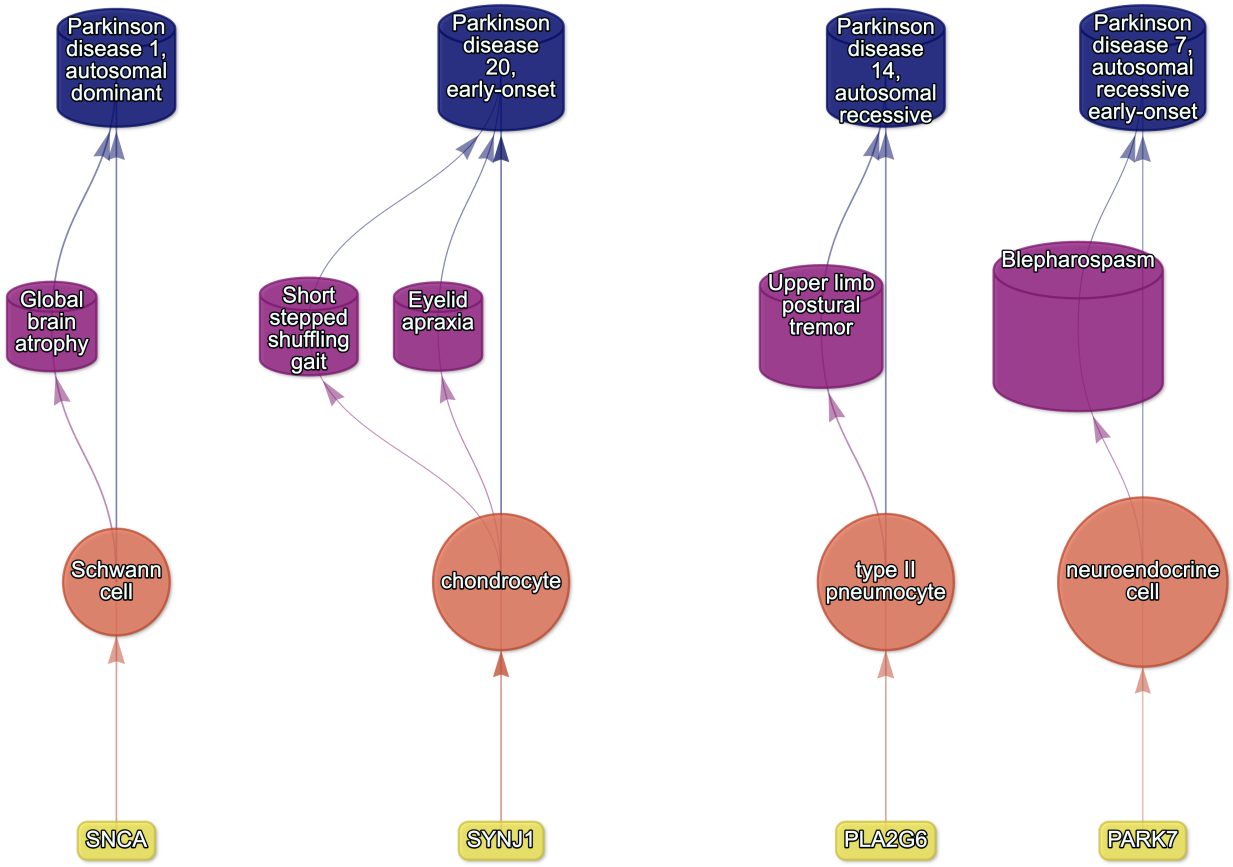

Figure S14: **Causal multi-scale network for phenotypes associated with various subtypes of Parkinson's disease.**

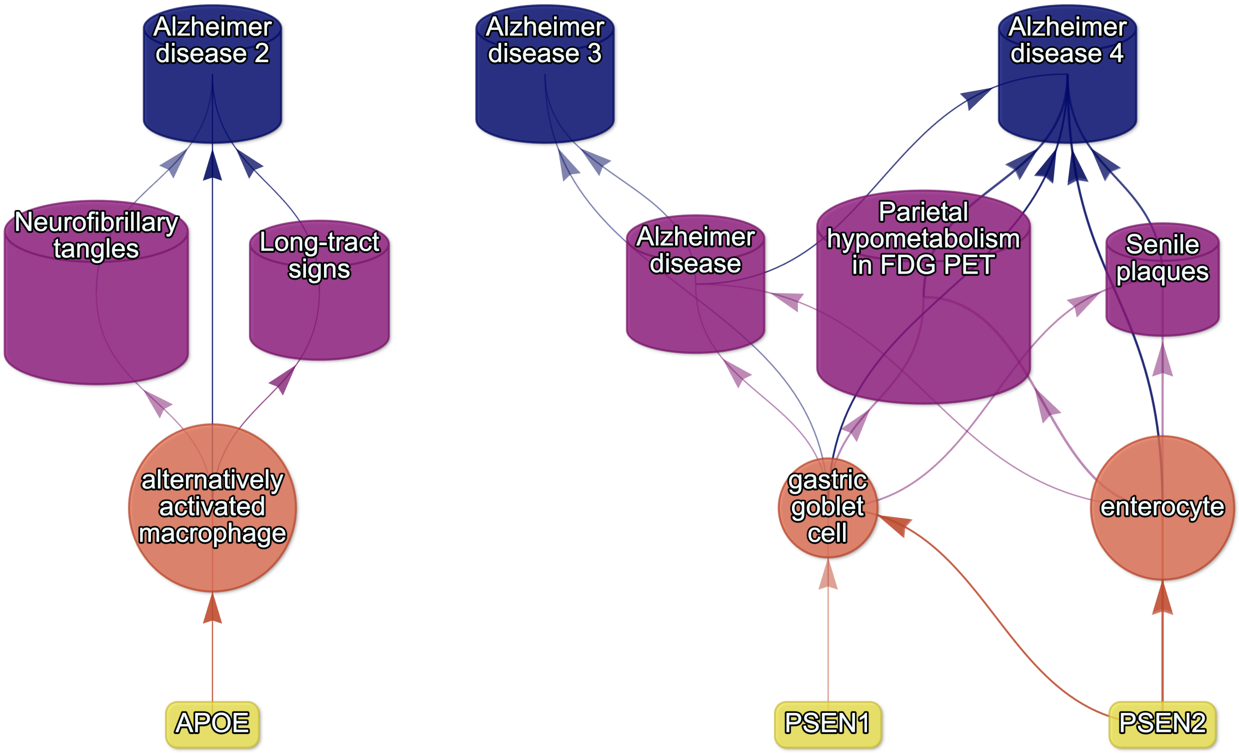

Figure S15: **Causal multi-scale network for phenotypes associated with various subtypes of Alzheimer's disease.**

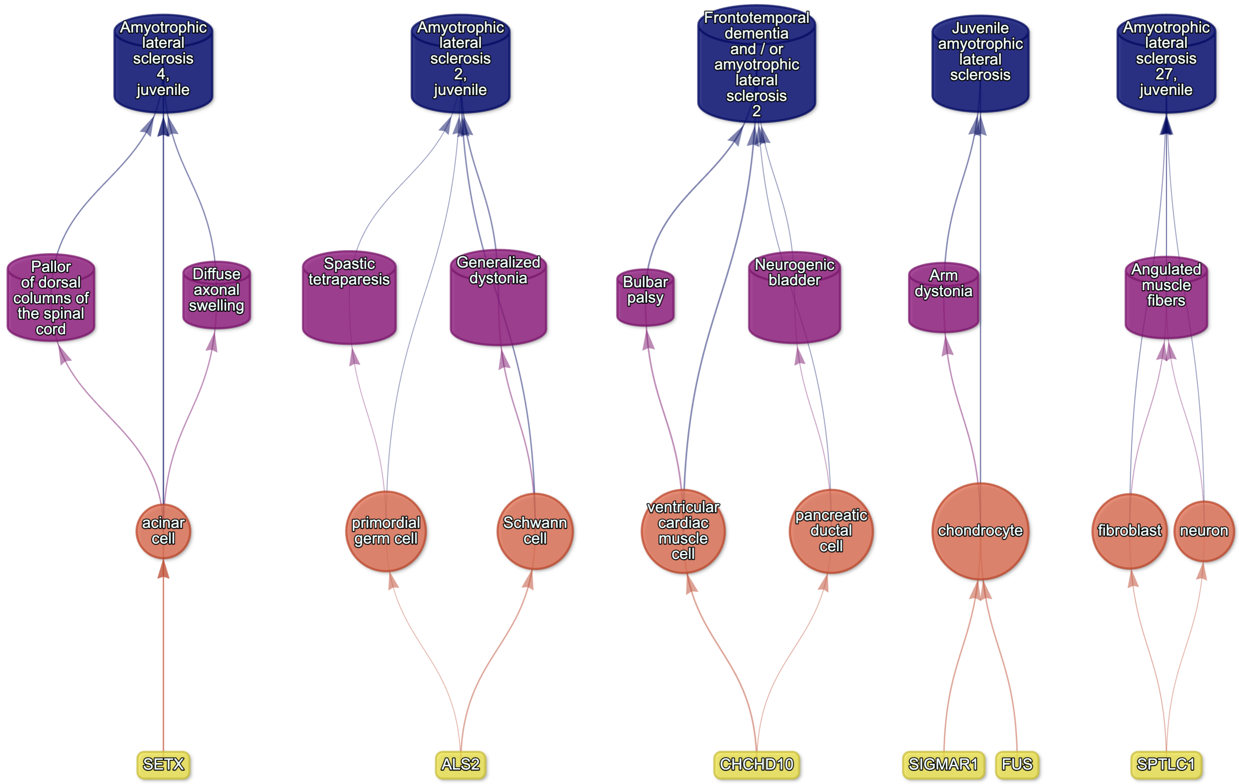

Figure S16: **Causal multi-scale network for phenotypes associated with Amyotrophic Lateral Sclerosis (ALS).**

1040 ## Supplementary Tables

Table S1: **Mappings between HPO phenotypes and other medical ontologies.** "source" indicates the medical ontology and "distance" indicates the cross-ontology distance. "source terms" and "HPO terms" indicates the number of unique IDs mapped from the source ontology and HPO respectively. "mappings" is the total number of cross-ontology mappings within a given distance. Some IDs may have more than one mapping for a given source due to many-to-many relationships.

source	distance	source terms	HPO terms	mappings
ICD10	2	25	23	25
ICD10	3	839	876	1170
ICD9	1	21	21	21
ICD9	2	434	306	462
ICD9	3	1052	920	1816
SNOMED	1	4413	3483	4654
SNOMED	2	75	21	78
SNOMED	3	1796	833	9605
UMLS	1	12898	11601	13049
UMLS	2	140	113	142
UMLS	3	1871	1204	11021

Table S3: **Description of each filtering step performed in the multi-scale therapeutic target prioritisation pipeline.** 'level' indicates the biological scale at which the step is applied to.

level	step	description
NA	1. start	NA
Cell type	2. q threshold	Keep only cell type-phenotype association results at q<=0.05.
Phenotype	3. keep descendants	Remove phenotypes belonging to a certain branch of the HPO, as defined by an ancestor term.
Phenotype	4. info content threshold	Keep only phenotypes with a minimum information criterion score (computed from the HPO).
Phenotype	5. severity threshold	Keep only phenotypes with mean Severity equal to or below the threshold.
Symptom	6. pheno frequency threshold	Keep only phenotypes with mean frequency equal to or above the threshold (i.e. how frequently a phenotype is associated with any diseases in which it occurs).
Gene	7. symptom gene overlap	Ensure that genes nominated at the phenotype-level also appear in the genes overlapping at the cell type-specific symptom-level.
Gene	8. evidence score threshold	Remove genes that are below an aggregate phenotype-gene evidence score threshold.

Table S3: **Description of each filtering step performed in the multi-scale therapeutic target prioritisation pipeline.** ‘level’ indicates the biological scale at which the step is applied to.

level	step	description
Gene	9. add driver genes	Keep only genes that are driving the association with a given phenotype (inferred by the intersection of phenotype-associated genes and gene with high-specificity quantiles in the target cell type).
Symptom	10. symptom intersection threshold	Minimum proportion of genes overlapping between a symptom gene list (phenotype-associated genes in the context of a particular disease) and the phenotype-cell type association driver genes.
Gene	11. gene frequency threshold	Keep only genes at or above a certain mean frequency threshold (i.e. how frequently a gene is associated with a given phenotype when observed within a disease).
Phenotype	12. prune ancestors	Remove redundant ancestral phenotypes when at least one of their descendants already exist.
All	13. top n	Only return the top N targets per variable group (specified with the “group_vars” argument). For example, setting “group_vars” to “hpo_id” and “top_n” to 1 would only return one target (row) per phenotype ID after sorting.
NA	14. end	NA

Table S2: **Summary statistics of enrichment results stratified by single-cell atlas.** Summary statistics at multiple levels (tests, cell types, phenotypes, diseases, cell types per phenotype, phenotypes per cell type) stratified by the single-cell atlas that was used as a cell type signature reference (Descartes Human or Human Cell Landscape).

	DescartesHuman	HumanCellLandscape	all
tests significant	19,929	26,585	46,514
tests	848,078	1,358,916	2,206,994
tests significant (%)	2.35	1.96	2.11
cell types significant	77	124	201
cell types	77	124	201
cell types significant (%)	100	100	100
phenotypes significant	7,340	9,049	9,575
phenotypes tested	11,014	10,959	11,028
phenotypes	11,047	11,047	11,047
phenotypes significant (%)	66.4	81.9	86.7
diseases significant	8,628	8,627	8,628
diseases	8,631	8,631	8,631
diseases significant (%)	100	100	100
cell types per phenotype (mean)	1.81	2.43	4.22
cell types per phenotype (median)	1	2	3
cell types per phenotype (min)	0	0	0
cell types per phenotype (max)	31	28	59
phenotypes per cell type (mean)	259	214	231
phenotypes per cell type (median)	252	200	209
phenotypes per cell type (min)	71	57	57
phenotypes per cell type (max)	696	735	735

Table S4: **Cross-ontology mappings between HPO and CL branches.** The last two columns represent the number of cell types that were overrepresented in the on-target HPO branch and the total number of cell types in that branch. A disaggregated version of this table with all descendant cell type names is available in Table S6.

HPO branch	Pheno-types (total)	CL branch	Cell types (over-represented)	Cell types (total)
Abnormality of the cardiovascular system	673	cardiocyte	5	6
Abnormality of the endocrine system	291	endocrine cell	3	4
Abnormality of the eye	721	photoreceptor cell/retinal cell	5	5
Abnormality of the immune system	255	leukocyte	14	14
Abnormality of the musculoskeletal system	2155	cell of skeletal muscle/chondrocyte	4	4
Abnormality of the nervous system	1647	neural cell	17	24
Abnormality of the respiratory system	292	respiratory epithelial cell/epithelial cell of lung	3	3

Table S5: **Encodings for GenCC evidence scores.** Assigned numeric values for the GenCC evidence levels.

classification_curie	classification_title	encoding
GENCC:100001	Definitive	6
GENCC:100002	Strong	5
GENCC:100003	Moderate	4
GENCC:100009	Supportive	3
GENCC:100004	Limited	2
GENCC:100005	Disputed Evidence	1
GENCC:100008	No Known Disease Relationship	0
GENCC:100006	Refuted Evidence	0

Table S6: **On-target cell types for each Human Phenotype Ontology (HPO) ancestral branch.** Cell type-phenotype branch pairings were manually curated by comparing high-level HPO terms to terms within the Cell Ontology (CL). Each HPO branch is shown as bolded row dividers. Ancestral CL branch names are shown in the first column, along with the specific CL names and IDs.

CL branch	CL name	CL ID
**Abnormality of the cardiovascular system**		
cardiocyte	cardiac muscle cell	CL:0000746
cardiocyte	regular atrial cardiac myocyte	CL:0002129
cardiocyte	endocardial cell	CL:0002350
cardiocyte	epicardial adipocyte	CL:1000309
cardiocyte	ventricular cardiac muscle cell	CL:2000046
**Abnormality of the endocrine system**		
endocrine cell	endocrine cell	CL:0000163
endocrine cell	neuroendocrine cell	CL:0000165
endocrine cell	chromaffin cell	CL:0000166
**Abnormality of the eye**		
photoreceptor cell / retinal cell	photoreceptor cell	CL:0000210
photoreceptor cell / retinal cell	amacrine cell	CL:0000561
photoreceptor cell / retinal cell	Mueller cell	CL:0000636
photoreceptor cell / retinal cell	retinal pigment epithelial cell	CL:0002586
**Abnormality of the immune system**		
leukocyte	T cell	CL:0000084
leukocyte	mature neutrophil	CL:0000096
leukocyte	mast cell	CL:0000097
leukocyte	microglial cell	CL:0000129
leukocyte	professional antigen presenting cell	CL:0000145
leukocyte	macrophage	CL:0000235
leukocyte	B cell	CL:0000236
leukocyte	dendritic cell	CL:0000451
leukocyte	monocyte	CL:0000576
leukocyte	plasma cell	CL:0000786
leukocyte	alternatively activated macrophage	CL:0000890
leukocyte	thymocyte	CL:0000893
leukocyte	innate lymphoid cell	CL:0001065
**Abnormality of the musculoskeletal system**		
cell of skeletal muscle / chondrocyte	chondrocyte	CL:0000138
cell of skeletal muscle / chondrocyte	cell of skeletal muscle	CL:0000188
cell of skeletal muscle / chondrocyte	skeletal muscle satellite cell	CL:0000594
**Abnormality of the nervous system**		
neural cell	bipolar neuron	CL:0000103
neural cell	granule cell	CL:0000120
neural cell	Purkinje cell	CL:0000121
neural cell	glial cell	CL:0000125
neural cell	astrocyte	CL:0000127
neural cell	oligodendrocyte	CL:0000128
neural cell	microglial cell	CL:0000129
neural cell	neuroendocrine cell	CL:0000165
neural cell	chromaffin cell	CL:0000166
neural cell	photoreceptor cell	CL:0000210
neural cell	inhibitory interneuron	CL:0000498
neural cell	neuron	CL:0000540
neural cell	neuronal brush cell	CL:0000555
neural cell	amacrine cell	CL:0000561
neural cell	GABAergic neuron	CL:0000617
neural cell	Mueller cell	CL:0000636
neural cell	glutamatergic neuron	CL:0000679
neural cell	retinal ganglion cell	CL:0000740
neural cell	retina horizontal cell	CL:0000745
neural cell	Schwann cell	CL:0002573
neural cell	retinal pigment epithelial cell	CL:0002586
neural cell	visceromotor neuron	CL:0005025
neural cell	sympathetic neuron	CL:0011103
**Abnormality of the respiratory system**		
respiratory epithelial cell / epithelial cell of lung	type II pneumocyte	CL:0002063
respiratory epithelial cell / epithelial cell of lung	epithelial cell of lower respiratory tract	CL:0002632

Table S7: **Results of permutation tests evaluating relationships between phenotype information content and gene number, cell type number, and effect size.** Correlations between phenotypes and cell types may not be independent due to the hierarchical nature of the HPO. To account for this, for each pair of variables we computed the observed correlation (*observed cor*), the mean null correlation (*null cor*) from 1000 permutations, and the 95% confidence interval of the null correlations (*null CI [lower, upper]*).

X	y	observed p	observed cor	empirical p	null cor	null CI [lower, upper]
info_content	cell types	6.91e-62	-0.61	2.23e-308	1.99e-03	[-0.07, 0.08]
info_content	estimate	2.23e-308	0.21	2.23e-308	1.18e-04	[-0.01, 0.01]
info_content	genes	2.23e-308	-0.58	2.23e-308	-1.67e-06	[0, 0]

Table S8: **Results of permutation tests evaluating phenotype-cell type association consistency across cell type datasets (Human Cell Landscape vs. Descartes Human; i.e. inter-CTD) and across developmental stages (foetal vs. Adult; i.e. intra-CTD).** To account for the non-independence between phenotypes, we computed empirical p-values for each Pearson correlation comparison over 1,000 permutations. Here, we report observed correlation (*observed cor*), the mean null correlation (*null cor*), 95% confidence interval of the null correlations (*null CI [lower, upper]*). "sig." indicates that only association tests with FDR<0.05 were included in the correlation.

comparison	observed p	observed cor	empirical p	null cor	null CI [lower, upper]
Inter-CTD: estimate (all)	0e+00	0.58	2e-308	2e-05	[-0.01, 0]
Inter-CTD: estimate (sig.)	1e-95	0.73	2e-308	-7e-04	[-0.08, 0.09]
Intra-CTD: estimate (all)	0e+00	0.44	2e-308	3e-05	[0, 0]
Intra-CTD: estimate (sig.)	4e-92	0.40	2e-308	4e-04	[-0.04, 0.04]

Table S9: **Some HPO phenotype categories are more biased towards foetal- or adult- versions of the same cell type.** We took the top 50 phenotypes with the greatest bias towards foetal-cell type associations ("Foetal-biased") and the greatest bias towards adult-cell type associations ("Adult-biased") and fed each list of terms into ontological enrichment tests to get a summary of the representative HPO branches for each group. The phenotypes most biased towards associations with only the foetal versions of cell type and those biased towards the adult versions of cell types are shown. "FDR" is the False Discovery Rate-adjusted p-value from the enrichment test, "log2-fold enrichment" is the log2 fold-change from the enrichment test, and "depth" is the depth of the enriched HPO term in the ontology.

term	name	FDR	log2-fold enrichment	depth
**Foetal-biased**				
HP:0005105	Abnormal nasal morphology	7.80e-06	4.5	6
HP:0010938	Abnormal external nose morphology	4.19e-05	5.4	7
HP:0000366	Abnormality of the nose	4.28e-05	3.8	5
HP:0000055	Abnormal female external genitalia morphology	6.32e-04	5.2	6
HP:0000271	Abnormality of the face	1.01e-03	1.9	4
HP:0000234	Abnormality of the head	1.59e-03	1.7	3
HP:0000152	Abnormality of head or neck	1.85e-03	1.6	2
HP:0010460	Abnormality of the female genitalia	3.27e-02	2.8	5
HP:0000811	Abnormal external genitalia	3.27e-02	2.8	5
HP:0000078	Abnormality of the genital system	3.27e-02	1.9	3
**Adult-biased**				
HP:0010647	Abnormal elasticity of skin	1.13e-05	6.0	5
HP:0008067	Abnormally lax or hyperextensible skin	2.04e-04	6.0	6
HP:0011121	Abnormal skin morphology	4.06e-04	2.4	4
HP:0000951	Abnormality of the skin	1.76e-03	2.1	3
HP:0001574	Abnormality of the integument	1.26e-02	1.6	2
HP:0001626	Abnormality of the cardiovascular system	2.39e-02	1.4	2
HP:0030680	Abnormal cardiovascular system morphology	2.39e-02	1.7	3
HP:0025015	Abnormal vascular morphology	3.99e-02	1.9	4
HP:0030962	Abnormal morphology of the great vessels	3.99e-02	2.7	6

Table S10: **Examples of specific phenotypes that are most biased towards associations with only the foetal versions of cell types ("Foetal-biased") and those biased towards the adult versions of cell types ("Adult-biased").** "p-value difference" is the difference in the association p-values between the foetal and adult version of the equivalent cell type (foetal-adult bias : $p_{adult} - p_{foetal} = \Delta p \in [-1, 1]$).

HPO name	HPO ID	CL ID	CL name	p-value difference
**Foetal-biased**				
Short middle phalanx of the 2nd finger	HP:0009577	CL:0000138	chondrocyte	0.99
Abnormal morphology of the nasal alae	HP:0000429	CL:0000057	fibroblast	0.95
Abnormal labia minora morphology	HP:0012880	CL:0000499	stromal cell	0.94
Acromesomelia	HP:0003086	CL:0000138	chondrocyte	0.93
Left atrial isomerism	HP:0011537	CL:0000163	endocrine cell	0.92
Fixed facial expression	HP:0005329	CL:0000499	stromal cell	0.92
Migraine without aura	HP:0002083	CL:0000163	endocrine cell	0.92
Truncal ataxia	HP:0002078	CL:0000163	endocrine cell	0.92
Anteverted nares	HP:0000463	CL:0000057	fibroblast	0.91
Short 1st metacarpal	HP:0010034	CL:0000138	chondrocyte	0.90
**Adult-biased**				
Symblepharon	HP:0430007	CL:0000138	chondrocyte	-0.97
Abnormally lax or hyperextensible skin	HP:0008067	CL:0000057	fibroblast	-0.94
Reduced bone mineral density	HP:0004349	CL:0000057	fibroblast	-0.94
Paroxysmal supraventricular tachycardia	HP:0004763	CL:0000138	chondrocyte	-0.93
Lack of skin elasticity	HP:0100679	CL:0000057	fibroblast	-0.92
Excessive wrinkled skin	HP:0007392	CL:0000057	fibroblast	-0.91
Bruising susceptibility	HP:0000978	CL:0000057	fibroblast	-0.91
Corneal opacity	HP:0007957	CL:0000057	fibroblast	-0.90
Broad skull	HP:0002682	CL:0000138	chondrocyte	-0.90
Emphysema	HP:0002097	CL:0000057	fibroblast	-0.89

Table S11: **Hypergeometric test results evaluating the enrichment of existing therapy targets documented in the Therapeutic Target Database (TTD) amongst our prioritised targets.** Results are shown separately for gene therapies and all therapies. Column keys: universe=gene universe size, p=hypergeometric test p-value, OR=odds ratio, NPV=negative predictive value, PPV=positive predictive value, FDR=false discovery rate, TP=true positives, FP=false positives, FN=false negatives, TN=true negatives.

status	overlap	universe	p	OR	sensitivity	specificity	PPV	NPV	FDR	TP	FP	FN	TN
**Gene therapies**													
nonfailed	65	5013	5.56e-05	3.00	0.83	0.38	0.02	0.99	0.98	65	3083	13	1852
failed	0	5013	1.00e+00	0.00	NaN	0.37	0.00	1.00	1.00	0	3148	0	1865
**All therapies**													
nonfailed	591	6432	1.00e+00	0.23	0.26	0.39	0.19	0.49	0.81	591	2557	1664	1620
failed	125	6432	4.44e-23	0.36	0.27	0.49	0.04	0.90	0.96	125	3023	335	2949
